# Circadian regulation of lung repair and regeneration

**DOI:** 10.1172/jci.insight.164720

**Published:** 2023-08-22

**Authors:** Amruta Naik, Kaitlyn M. Forrest, Oindrila Paul, Yasmine Issah, Utham K. Valekunja, Soon Y. Tang, Akhilesh B. Reddy, Elizabeth J. Hennessy, Thomas G. Brooks, Fatima Chaudhry, Apoorva Babu, Michael Morley, Jarod A. Zepp, Gregory R. Grant, Garret A. FitzGerald, Amita Sehgal, G. Scott Worthen, David B. Frank, Edward E. Morrisey, Shaon Sengupta

**Affiliations:** 1 Children’s Hospital of Philadelphia, Philadelphia, Pennsylvania, USA.; 2 Systems Pharmacology, Perelman School of Medicine, Philadelphia, Pennsylvania, USA.; 3 Institute of Translational Medicine and Therapeutics (ITMAT), and; 4 Chronobiology and Sleep Institute, University of Pennsylvania, Philadelphia, Pennsylvania, USA.; 5 Penn-CHOP Lung Biology Institute,; 6 Department of Pediatrics,; 7 Department of Genetics,; 8 Department of Neuroscience, and; 9 Department of Cell and Developmental Biology, Perelman School of Medicine, University of Pennsylvania, Philadelphia, Pennsylvania, USA.

**Keywords:** Pulmonology, Virology, Influenza, Mouse models, Respiration

## Abstract

Optimal lung repair and regeneration are essential for recovery from viral infections, including influenza A virus (IAV). We have previously demonstrated that acute inflammation and mortality induced by IAV is under circadian control. However, it is not known whether the influence of the circadian clock persists beyond the acute outcomes. Here, we utilize the UK Biobank to demonstrate an association between poor circadian rhythms and morbidity from lower respiratory tract infections, including the need for hospitalization and mortality after discharge; this persists even after adjusting for common confounding factors. Furthermore, we use a combination of lung organoid assays, single-cell RNA sequencing, and IAV infection in different models of clock disruption to investigate the role of the circadian clock in lung repair and regeneration. We show that lung organoids have a functional circadian clock and the disruption of this clock impairs regenerative capacity. Finally, we find that the circadian clock acts through distinct pathways in mediating lung regeneration — in tracheal cells via the Wnt/β-catenin pathway and through IL-1β in alveolar epithelial cells. We speculate that adding a circadian dimension to the critical process of lung repair and regeneration will lead to novel therapies and improve outcomes.

## Graphical abstract

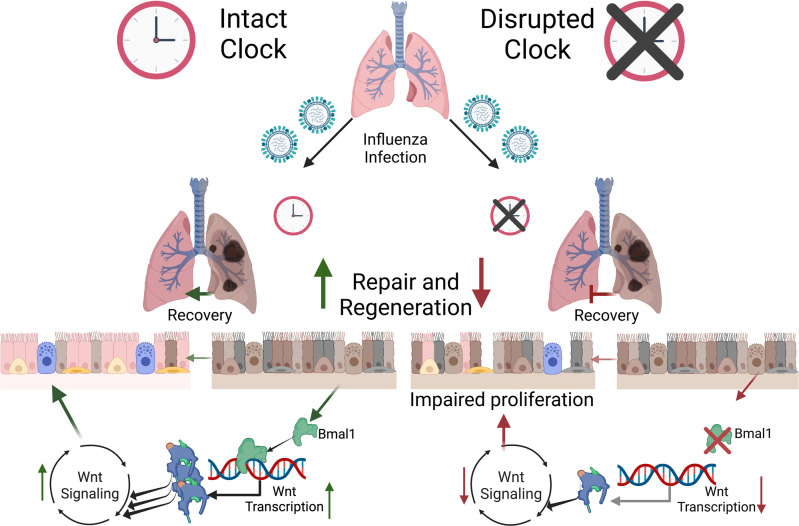

## Introduction

Circadian rhythms provide an anticipatory system by temporally compartmentalizing physiological processes and thus allowing organisms to maintain homeostasis in the face of changes in their environment. At the molecular level, these cell-autonomous clocks comprise transcriptional-translational feedback loops of core clock genes that drive the rhythmic fluctuations of many cellular processes through direct or indirect effects on downstream targets (1). Circadian profiling of the lung transcriptome reveals that most physiological pathways, including those involved in the regulation of lung injury and repair, are characterized by rhythmically expressed genes (2). Furthermore, lung inflammation has been shown to be under circadian control in several injury models (3–5). We have previously demonstrated that disruption of the circadian clock leads to higher acute mortality from influenza A virus (IAV) infection in mice independently of viral burden (6). We also found that lower-amplitude circadian rhythms in adults was associated with higher likelihood of hospitalization from lung infections. However, this was based on actigraphy either before or after the diagnosis of infection, which hampered our ability to suggest causality (7).

Recovery from IAV infection is dependent not only on inflammation, but also the ability of the host to repair and regenerate injured lungs. Although we and others have investigated the role of the clock in acute inflammation, its effect on lung repair and regeneration is not known. A few studies have suggested that the circadian clock enhances tissue regeneration in skin and in intestines, driven by actin assembly in skin fibroblasts or regulation of cell proliferation in both skin and intestines (8–10). However, both skin and intestines have very high cellular turnover rates at baseline and are constantly proliferating and differentiating. The uninjured lung, on the other hand, is quiescent. However, following acute inflammation induced by IAV infection, the lung enters a state of rapid proliferation (11). Whether the circadian clock improves lung proliferation following IAV is not known. Our previous work supports the importance of at least 2 pulmonary epithelial cells, Sftpc^+^ alveolar type 2 (AT2) cells (12) and Scgb1a1^+^ club cells (6) in the circadian control of outcomes from IAV. Acute mortality and lung injury were worse when the circadian clock was specifically disrupted in Sftpc^+^ or Scgb1a1^+^ cells. Interestingly, both these cell types are central to lung repair and regeneration following IAV infection (13, 14). Thus, we hypothesized that a functioning circadian clock improves long-term recovery from IAV through lung repair and regenerative pathways.

Here, we utilize a combination of epidemiological data from the United Kingdom (UK) Biobank and mechanistic mouse models to determine whether disruption of the circadian clock worsens long-term outcomes following lung infection. We quantify both lung inflammation in the chronic phase of lung recovery and employ lung organoid assays to determine the extent to which the circadian clock contributes to lung regeneration independently of clock-gated inflammation. We demonstrate, for the first time to our knowledge, that lung organoids have their own intrinsic clock, and that disruption of the circadian clock impairs lung regenerative responses. We use a combination of tissue-specific, constitutive, and inducible *Bmal1^–/–^* and constitutive *Cry1^–/–^* *Cry2^–/–^* models, epithelial cell–specific clock mutants, and single-cell RNA sequencing (scRNA-seq) to demonstrate that the clock aids lung repair and regeneration by controlling proliferation during recovery from IAV infection. Specifically, we identify circadian control of the Wnt/β-catenin pathway as a mechanism for clock-gated cell proliferation.

## Results

### Individuals with poor circadian rhythms are more likely to have worse outcomes from pneumonia.

**Figure 1:**
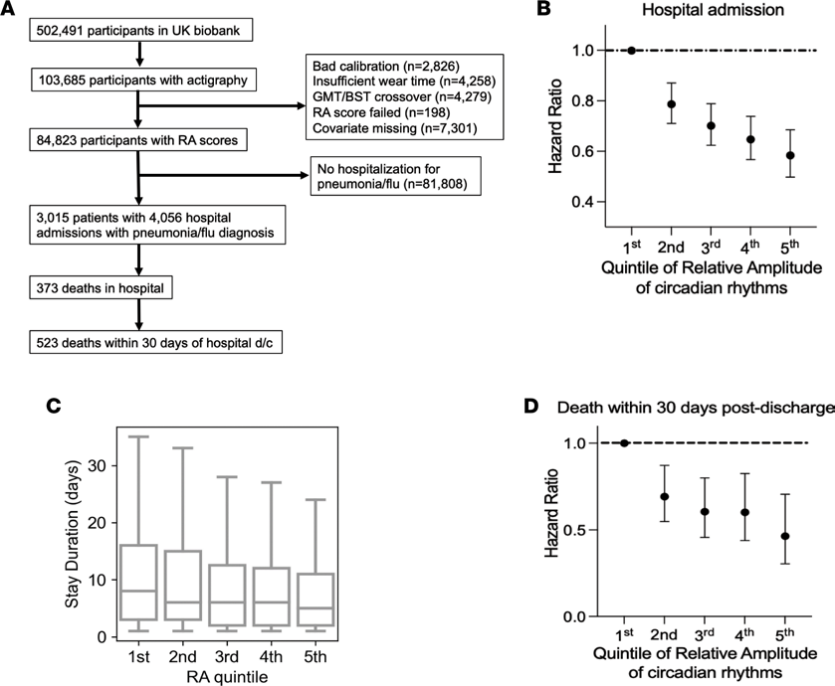
UK Biobank analyses. (**A**) Description of study cohort from UK Biobank. (**B**) Hazard ratio for the risk of hospitalization for respiratory infections across the quintiles of relative amplitude (RA) of actigraphy as a measure of circadian rhythms. The lowest quintile represents individuals with the poorest circadian rhythms and serves as the comparison group. (**C**) Length of hospital stay defined across the quintiles of RA scores (median, IQR). (**D**) Hazard ratio of the risk of death within 30 days after discharge from above-defined hospitalization. All hazard ratios were adjusted for cofounding factors that included, age, sex, HbA1c, cancer diagnosis, FEV1, BMI, smoking status, history of previous respiratory illness requiring hospitalization, overall physical activity level, use of bronchodilators and inhaled corticosteroids, and self-rated overall health.

We have previously shown that poor circadian rhythmicity (as reflected by low “rhythm amplitude” or RA) was associated with an increased risk of hospital admission for influenza and other lower respiratory tract infections (7). RA was calculated based on hand-worn actigraphy data and has been previously validated for the study of circadian rhythms (15, 16). However, in our previous work (7), the association between low-amplitude circadian rhythms and respiratory outcomes was reported based on actigraphy data collected before or after the outcome of interest, thus limiting our understanding of poor rhythms as a risk factor for outcomes. Here, we specifically investigated whether poor circadian rhythms predicted worse respiratory outcomes. Actigraphy data along with all covariates of interest were available for 84,823 individuals. Of these, 3,015 patients eventually required hospitalization for respiratory infections, including influenza (Figure 1A). On average, actigraphy data were collected 3.9 years before hospitalization in this cohort (Supplemental Figure 1A; supplemental material available online with this article; https://doi.org/10.1172/jci.insight.164720DS1). We divided the RA data into quintiles, such that the individuals in the first quintile had the lowest RA scores and poorest measures of circadian rhythm. The distribution of common confounders across the RA quintiles is depicted in Supplemental Figure 1B. Outcomes studied included need for hospitalization for respiratory infection, duration of hospitalization, and death during hospitalization as surrogates for poor lung repair and severe lung inflammation following infection. Individuals in the highest quintile of RA score had only 58% (95% CI, 48%–67%) of the rate of hospitalization for pneumonia or influenza compared with those in the lowest quintile (*P* = 3 × 10^–12^, *n* = 84,823 individuals with hospitalizations) adjusting for age, sex, HbA1c, cancer diagnosis, FEV_1_, BMI, smoking status, history of previous respiratory illness requiring hospitalization, overall physical activity level, use of bronchodilators and inhaled corticosteroids, as well as self-rated overall health (Figure 1B). Moreover, those in this highest quintile experienced only 49% (95% CI, 30%–81%) of the rate of in-hospital death for pneumonia or flu (*P* = 0.005, *n* = 373 deaths). The duration of hospital stay also decreased with increasing RA scores, with those in the lowest quintile staying 2 additional days beyond those in the highest quintile (median [IQR] in first versus the fifth quintile was 7 [12] and 5 [9], respectively), with an overall faster discharge in those with robust circadian rhythms (*P* = 0.006, *n* = 4078 hospitalizations; Figure 1C).

Finally, even after discharge home, those with poorer circadian rhythms in the years preceding the hospitalization continued to experience adverse effects consistent with poorer lung repair. The 30-day postdischarge mortality was highest among those with poorest circadian rhythms (HR = 0.44 [95% CI, 0.29–0.67], *P* = 1 × 10^–4^, *n* = 523 deaths; Figure 1D). For the above analyses, the RA score had larger effect size than overall physical activity levels, medication use, and smoking, underscoring the importance of amplitude of circadian rhythms as a risk factor for respiratory morbidity that is independent of overall physical activity or exercise (Supplemental Figure 1C).

### Temporal gating of alveolar damage on day 30 after recovery is controlled by the circadian clock.

**Figure 2:**
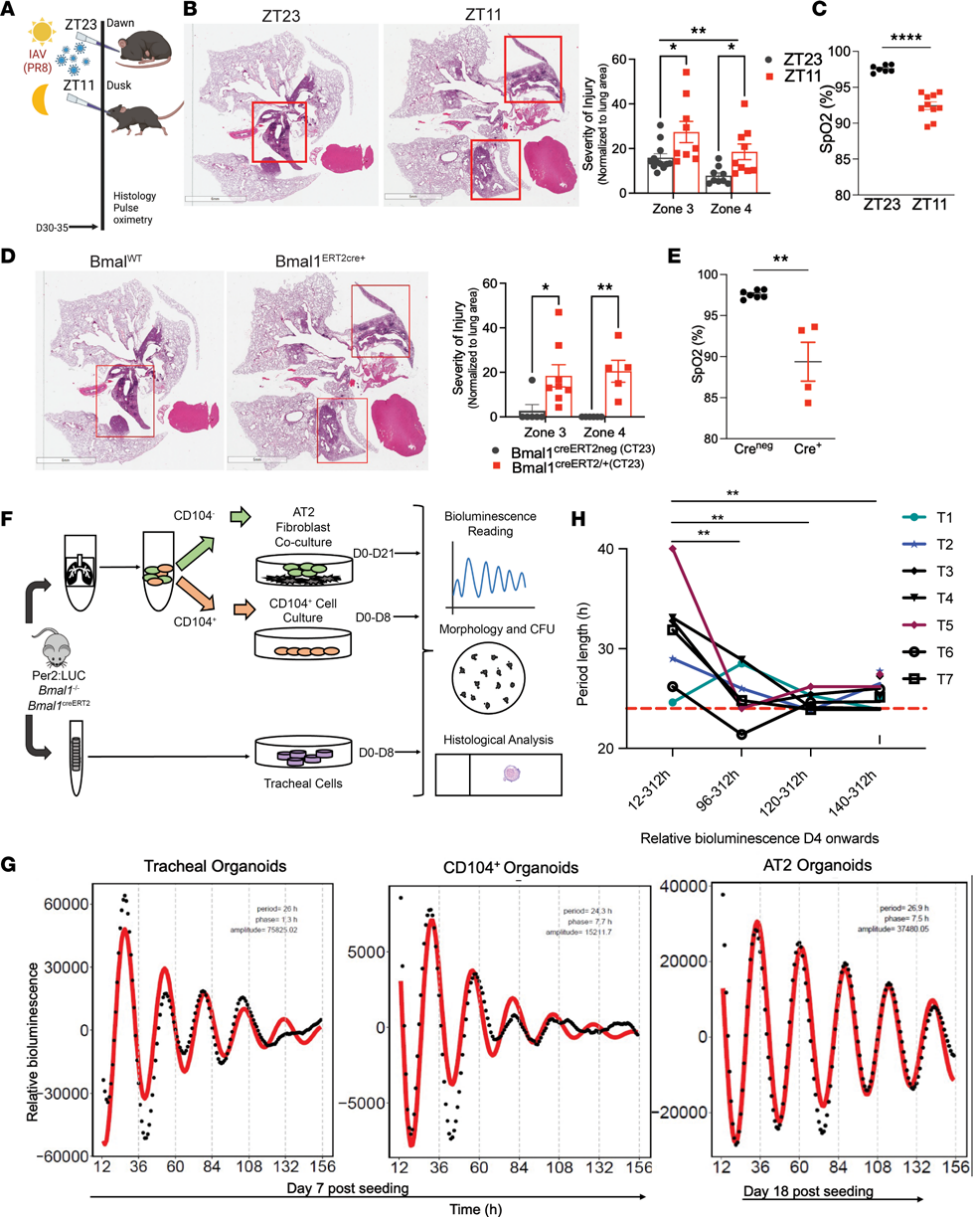
Infection time of day affects long-term lung repair and regeneration. (**A**) Schematic of experimental design: Mice were infected with influenza A virus (IAV) at either dawn (ZT23/rest phase) or dusk (ZT11/active phase). (**B**) Left: Representative micrographs of H&E-stained lung sections 30 days post infection (p.i.). The red box denotes areas with maximal alveolar destruction — zones 3 and 4 in our scoring system. Scale bars: 5–6 mm. Right: Quantification of the severity of lung injury. **P = 0.0029 for time of infection by 2-way ANOVA. *P = 0.016 for zone 3 and **P = 0.04 for zone 4 on multiple comparison with Bonferroni’s correction. (**C**) Pulse oximetry on day 35 p.i. ****P < 0.0001 by unpaired 2-tailed *t* test. (**D**) Left: Representative micrographs of H&E-stained lung sections from *Bmal1^creERT2/+^* and cre^neg^ littermates 30 days p.i. Scale bars: 5–6 mm. Right: Quantification. *P = 0.0028 for time of infection, 2-way ANOVA. *P = 0.0171 for zone 3 and **P = 0.0056 for zone 4 on multiple comparison with Bonferroni’s correction. Data were pooled from 2–4 independent experiments and are represented as mean ± SEM. (**E**) Pulse oximetry on day 35 p.i. **P = 0.0061 by Mann-Whitney test. (**F**) Experimental design for organotypic assays. (**G**) Representative tracing of relative bioluminescence of *Per2* expression in organoids grown from different levels of respiratory tree from *mPer2^Luc^* mice. (**H**) Relative bioluminescence recording of tracheal organoids 4 days after seeding. Organoid data are summarized from 3–5 independent experiments with at least 3 technical replicates/experiment. Two-way ANOVA with Tukey’s multiple-comparisons test. **P = 0.009, 0.004, 0.005; hours of bioluminescence **P = 0.0019.

We have previously shown that mice infected with IAV at dusk (ZT11) had a 3-fold higher mortality than littermates and cage mates infected at dawn (ZT23), and this time-of-day–specific protection was lost when the circadian clock is disrupted genetically in adulthood (6). (By circadian nomenclature, ZT0 refers to the time when lights turn on in a 12-hour light/dark cycle. Thus, ZT11 or dusk refers to the time just before the onset of the active phase and ZT23 refers to the time just before the onset of rest phase in mice who are nocturnal. The experiments in mutant mice are performed in constant darkness to avoid confounding effects of light. Under constant darkness, the times corresponding to dusk/ZT11 and dawn/ZT23 are referred to as CT11 and CT23, respectively). To determine whether the time of day at initial infection affects lung repair past the phase of acute injury and inflammation, we infected C57BL6/J mice with IAV (H1N1/PR8; sublethal dose) at ZT11 or ZT23 and harvested lungs 30 days post infection (p.i.) (Figure 2A). No significant mortality was noted at this dose in either group. Interestingly, we found that even 1 month after the initial infection, mice infected at ZT11 had significantly more lung damage and alveolar destruction than the group infected at ZT23. The scoring system divides the lung into 4 zones based on severity of the injury (17) and is detailed elsewhere in Methods and Supplemental Figure 2A. Zone 1 has no injury and zone 2 has mild interstitial thickening. Zone 3, which reflects substantial injury, was scored as 15.87% in ZT23 versus 27.37% in ZT11 (*P* = 0.007). Zone 4 or complete alveolar destruction: 10.04% in ZT23 versus 18.52% in ZT11 group (*P* = 0.02, Mann-Whitney test; Figure 2B). Even when we stratified by this by sex, the group infected at ZT23 continued to have better recovery and repair than the group infected at ZT11 (Supplemental Figure 2B). Supporting these histological findings, we found evidence of better gas exchange in the group infected at ZT23 than the ZT11 group, as seen on pulse oximetry performed on day 35 p.i. (Figure 2C). Lungs harvested form mice infected at ZT11 also showed more fibrosis (Supplemental Figure 3, A and B using modified Ashcroft’s scoring and using Picrosirius red staining in Supplemental Figure 3, C and D) (18). This was confirmed by a quantitative assay for soluble collagen (Supplemental Figure 3E). In areas of comparable damage in ZT11 and ZT23, KRT5 expression was similar. However, given that the lungs from the ZT11 group showed more areas of injury, lungs from this group had higher overall KRT5 expression (Supplemental Figure 3G). In animals in which the clock was disrupted in adulthood by inducing global deletion of the core clock gene *Bmal1* (*Bmal1^creERT/+^* versus control *Bmal1^creERTneg^*) had significantly worse injury and more lung fibrosis than cre^neg^ littermates (Figure 2D and Supplemental Figure 3F). *Bmal1^creERT/+^* mice also had significantly lower saturations than *Bmal1^creERTneg^* littermates, providing good physiological correlation for our histological findings (Figure 2E). Thus, the temporal protection from circadian rhythmicity persists well beyond the acute phase of inflammation and disruption of the circadian clock worsens histology several weeks following infection. We have previously reported that the group infected at ZT11 had more inflammation and immunopathology on days 2 and 6, despite being able to clear the infection successfully by day 8 to 10 p.i. (6). Therefore, one possibility is that the groups that do poorly have persistent and exaggerated clock-gated inflammation that continues to worsen the severity of lung injury even after the acute inflammation. The other possibility is that the circadian clock contributes to these long-term outcomes via lung repair and regenerative processes that are independent of the initial clock-controlled inflammatory response. These possibilities are not mutually exclusive and so we tested them both.

### Beyond the acute inflammatory phase, the clock directs only mild changes in lung inflammation.

To test whether the long-term temporal protection from IAV mediated via the circadian clock (as seen in Figure 2) is driven by regulation of inflammation, we enumerated immune cells in the lungs on days 10 and 30 p.i. We hypothesized that the ZT11 group will continue to show exaggerated inflammation relative to the ZT23 group even after the acute phase of the infection and that this would be evident on days 10 and 30 p.i. While we have previously demonstrated an early increased inflammation on days 2–6 p.i. (6), we found that by day 10 p.i., this had resolved and the total leukocytes were not increased in the group infected at ZT11 compared to ZT23 (Supplemental Figure 4A). While there was no difference in neutrophil or inflammatory monocyte populations at that time point, the ZT11 group had significantly higher tetramer^+^ CD8^+^ cells and a trend toward increasing vascular permeability on day 10 p.i. (Supplemental Figure 5, A and B). For evaluation 1 month p.i., we focused only on areas of substantial injury or total alveolar destruction (zones 3 and 4 respectively, as defined above). Despite very clear differences in alveolar injury (Figure 2B), there were only subtle differences in the lymphocytic and macrophage populations in the zones of alveolar damage. There were slightly more CD3^+^ (lymphocytes) cells in zone 3 but not zone 4 of injured lungs harvested from mice infected at ZT11; likewise, there was a slight predominance of F4/80^+^ nuclei in zone 4 but not in zone 3 at the same time point (Supplemental Figure 4, B–G). Even more intriguingly, we found that neither the lymphocytic nor macrophage infiltration was significantly higher in the *Bmal1^creERT/+^* mice than their cre^neg^ littermates (Supplemental Figure 4, H–K). Comparing the relatively subtle changes in inflammation on days 10 and 30 p.i. with the more dramatic lung injury on day 30 p.i. (Figure 2B, Supplemental Figure 2, B and C, and Supplemental Figure 3, A and B), we speculated that perhaps the latter reflects the combined effects of circadian clock–driven inflammation and lung repair. Therefore, we next investigated whether the circadian clock contributes to these long-term outcomes via lung repair and regenerative processes that are independent of the initial clock-controlled inflammatory response using lung organoid assays.

### Organoids derived from different levels of the respiratory tract display robust circadian rhythmicity.

To allow us to dissociate clock-driven lung inflammation from primary regenerative effects mediated by the circadian clock, we used organotypic assays. However, we first wanted to determine whether lung organoids maintained an active clock. While many studies have demonstrated the presence of a cell-autonomous clock active in in vitro cultures, evidence of the same in organotypic cultures is limited, mainly restricted to rapidly proliferating, intestinal organoids (9, 10, 19) or metabolically active, β (islet) cell organoids (20). We grew lung organoids from different levels of the respiratory epithelium, namely alveoli, small airways, and trachea of *mPer2^luc^* mice (21) (Figure 2D and Supplemental Figure 6, A–D).

Lung organoids from all these regions exhibited robust diurnal oscillations, even in the absence of synchronizing agents, for more than 108 hours for tracheal organoids (period: 26.76 ± 1 hours), greater than 108 hours for CD104^+^ organoids from smaller airways (period: 25.38 ± 0.97 hours), and greater than 132 hours for alveolar organoids (27.34 ± 0.18 hours) (Figure 2E and Supplemental Figure 6). Tracheal organoids mature by day 7 in matrigel, and the bioluminescence data in Figure 2E were recorded after this period of maturation. However, when the recording was started on day 4 after seeding, the period length was substantially longer and variable. As the tracheal organoids matured, the period length decreased to a “24-hour” period, consistent with circadian rhythmicity and was noted to be much less variable (Figure 2F). This effect was lost, and the period was closer to approximately 24 hours when a synchronizing agent was used (Supplemental Figure 6F). This suggests that maturation in organotypic cultures is paralleled by greater synchronization of their circadian clocks across the different cells within the 3D culture system. These results confirm the existence of cell-intrinsic clocks in lungs organoids and further support their use in studying the effect of the circadian rhythms on lung regeneration.

### Clock disruption reduces the regenerative potential of tracheal and distal lung basal cell organoids.

Next, we examined the role of the circadian clock in modulating the regenerative capacity of epithelial cells from all levels of the respiratory tree that were capable of self-renewal and/or differentiation (Figure 2D). Only cells capable of regeneration contribute to the growth of these self-organizing structures. These include AT2 cells, basal cells, and club cells. Although alveolar regeneration is key to repair and regeneration following IAV infection, several studies have shown that not only AT2 cells (13, 22) but also basal and club cells play a critical role in the repair process (23–26). As there are no established unique surface markers for sorting live club cells, we used the following strategy — organoids grown from the CD104^+^ epithelial cells reflect the regenerative capacity of a combination of club cells and basal cells from smaller airways, including a population of CD104^+^ lineage-negative (CD104^+^Lin^–^) epithelial progenitor and SOX2^+^Lin^–^ cells, both known for their role in lung repair (27, 28) (Supplemental Figure 4B). Tracheal organoids reflect the regenerative capacity of club cells and basal cells but from trachea/larger airways (Supplemental Figure 4, A and B), which have also been known to play an important role in lung regeneration (29).

**Figure 3:**
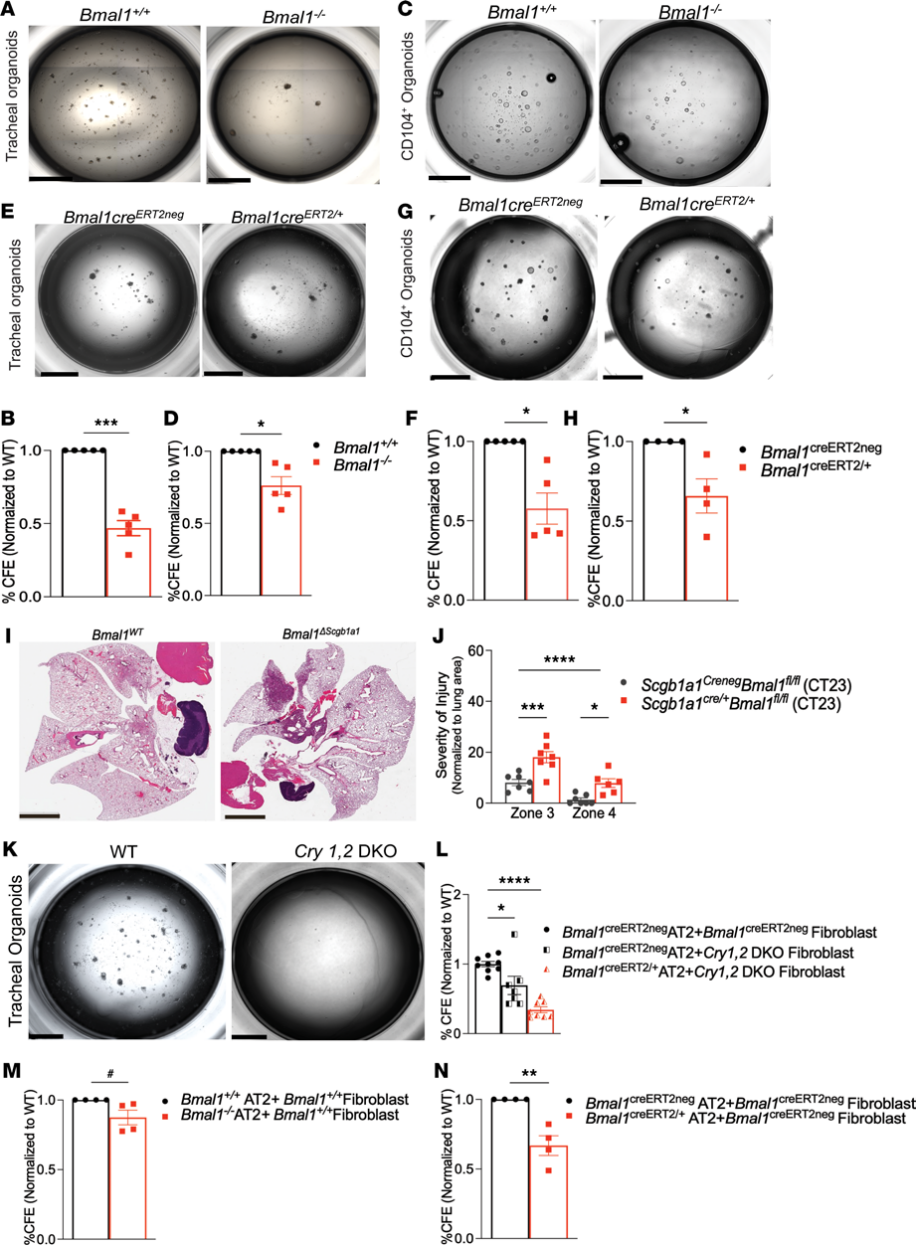
Deletion of *Bmal1* reduces regenerative capacity in lung organoids. Lungs from embryonic *Bmal1^–/–^* and their *Bmal1^+/+^* littermates were used for organotypic assays. For postnatal deletion of *Bmal1*, *Bmal1^creERT2/+^* and their cre^neg^ littermates were treated with tamoxifen at 8 weeks of age. Representative images of tracheal organoids from (**A**) *Bmal1^+/+^* and *Bmal1^–/–^*. (**B**) Regenerative capacity was quantified as colony-forming efficiency (CFE). (**C**) *Bmal1^creERT2neg^* and *Bmal1^creERT2/+^* mice. (**D**) Quantification (CFE). (**E**) Representative images of CD104^+^ distal lung cell organoids grown from *Bmal1^+/+^* (WT) and *Bmal1^–/–^* mice. (**F**) Quantification as CFE. (**G**) *Bmal1^creERT2neg^* and *Bmal1^creERT2/+^.* (**H**) Quantification as CFE. (**I**) Representative micrographs of H&E-stained lung sections from *Scgb1a1^Cre/+^* *Bmal1^fl/fl^* mice and *Scgb1a1^Creneg^* *Bmal1^fl/fl^* littermates, infected as in Figure 2A and recovered until day 30. Scale bars: 5 mm. (**J**) Quantification. Each data point represents an individual animal, and data were pooled from 2 independent experiments (*n* = 7–8 mice per circadian time point) 30 days p.i. ****P < 0.0001 for genotype by 2-way ANOVA; ***P = 0.0002 for zone 3; *P = 0.015 for zone 4 on multiple testing. (**K**) Representative images of tracheal organoids from *Cry1^–/–^* *Cry2^–/–^* (*Cry1,2*–DKO) mice. Scale bar: 2000 μm. (**L**) AT2 organoids cocultured with *Cry1,2*–DKO fibroblasts. (**M**) Embryonic and (**N**) postnatal *Bmal1*-knockout mice. Organoid experiments: Data pooled from 3–5 independent experiments with at least 3 technical replicates/experiment and expressed as mean ± SEM. **B**: ***P = 0.005; **D** and **F**: *P = 0.01; **H**: *P = 0.04; **J** and **L**: *P = 0.01, ****P = 0.0001. Ordinary 1-way ANOVA. **M**: ^#^*P* = 0.09; **N**: *P = 0.01. Unpaired 2-tailed *t* test with Welch’s correction.

We found that embryonic loss of *Bmal1* resulted in a 54% decrease in colony-forming efficiency (CFE) (Figure 3, A and B; *P* = 0.0005) of tracheal organoids. Similarly, loss of *Bmal1* also decreased the regenerative capacity of CD104^+^ organoids by 24% (Figure 3, C and D; *P* = 0.01) in comparison with organoids derived from the *Bmal1*-sufficient CD104^+^ lung cells. Although *Bmal1* is the only nonredundant core clock gene, the global phenotype is modulated by its noncircadian functions (30). If the regenerative loss seen in the embryonic knockout (KO) model is truly secondary to its circadian role, we argued that this phenotype should be preserved in a postnatal model wherein the clock has been disrupted in adulthood (~8 weeks of life).

Indeed, we found that even postnatal loss of *Bmal1* (*Bmal1^creERT2/+^*) decreased the regenerative capacity of tracheal organoids by 43% (Figure 3, E and F; *P* = 0.01) and of CD104^+^ organoids by 35% (Figure 3, G and H; *P* = 0.01) as compared with *Bmal1^creERT2neg^* organoids. To determine whether these findings from organoid models portend worse lung injury in vivo, we infected *Scgb1a1^cre/+^* *Bmal1^fl/fl^* mice, in which the clock has been selectively disrupted in club cells (Supplemental Figure 7A), and their cre^neg^ littermates with IAV at CT23 and recovered them to day 30 p.i. We have previously reported that deletion of *Bmal1* in Scgb1a1^+^ or club cells abrogates the time-of-day–specific protection in acute mortality and results in outcomes comparable to the WT animals infected at ZT11 (6). However, it is unknown whether this influences long-term outcomes. Here, we found that *Scgb1a1^cre/+^* *Bmal1^fl/fl^* mice had significantly worse alveolar injury than their cre^neg^ (1.35% zone 4 injury in cre^neg^ littermates vs. 7.9% in *Scgb1a1^cre/+^* *Bmal1^fl/fl^*; Figure 3, I and J). Although Gibbs et al. (5) have found different *Scgb1a1-Cre^+^* *Bmal1^fl/fl^* mice to have more neutrophils in their bronchoalveolar lavage, we did not find the bronchoalveolar lavage count to be higher overall in the *Scgb1a1-Cre^+^* *Bmal1^fl/fl^* mice compared to their cre^neg^ littermates (Supplemental Figure 7C). The lungs of the *Scgb1a1-Cre^+^* *Bmal1^fl/fl^* mice were not grossly distorted or inflamed (Supplemental Figure 7B). Thus, not only does clock disruption in club cells have lasting effects on lung repair and regeneration in vivo, but consistent results are obtained in organoid assays from smaller airways and trachea. They support the role of the clock in mediating lung injury and repair, above and beyond effects that are explained by clock-controlled acute inflammation.

To test whether this loss of regenerative capacity was *Bmal1* specific or broadly recapitulated in other models of clock disruption, we used lung cells from *Cry*1^–/–^ *Cry*2^–/–^ double KO (*Cry1*,*2*–DKO) mice. Tracheal organoids derived from *Cry1*,*2*–DKO mice show drastically reduced regenerative capacity, with hardly any organoids seen in the mutant (Figure 3, K and L). When comparing the 2 models of *Bmal1* deletion, we noted that the organoids derived from the embryonic *Bmal1* deletion model demonstrated a rather dysplastic appearance (Supplemental Figure 6, C and D). This was not present in organoids derived from the inducible model. Overall, the sizes of the organoids were comparable. Thus, while subtle differences in organoid appearance may be present in the 2 *Bmal1* deletion models, they remain comparable at least in regenerative efficiency.

Taken together, these findings confirm that an intact circadian clock improves lung recovery through enhanced regeneration, independently of the influence of clock-gated inflammation. To understand whether the poor regenerative capacity due to clock disruption is specific to epithelial cells in larger airways (tracheal cells and bronchial cells) or globally relevant to all cell types, we investigated the effect of *Bmal1* silencing on alveolar cells.

### Clock disruption reduces the regenerative capacity of alveolar cells.

We cultured CD45^–^CD31^–^EpCAM^+^CD104^–^ cells comprising both AT2 and AT1 cells (Supplemental Figure 6A) in organotypic assays. As described in the previous section, we used both the global embryonic and postnatal inducible *Bmal1*^–/–^ models. Lung fibroblasts from WT mice were used as feeder cells, to support AT2 cell self-renewal (22, 31).

There was a 13% decrease in regenerative capacity with embryonic deletion of *Bmal1* in AT2 cells supported by WT fibroblasts (Figure 3M and Supplemental Figure 7D). Interestingly, the loss of regenerative potential was more marked for alveolar organoids derived from alveolar cells following postnatal deletion of *Bmal1* than from those following the embryonic loss of *Bmal1*. There was a 37% reduction in regenerative capacity in *Bmal1^creERT2/+^* AT2 cells cocultured with WT feeder cells (Figure 3N and Supplemental Figure 7D). This effect was not limited to *Bmal1* deletion, and a similar deficiency was noticed when *Cry1,2–*DKO cells were used. Organoids growing using *Cry1,2*–DKO AT2 cells and WT fibroblasts showed dramatically reduced regenerative capacity, with hardly any organoids seen 18–21 days following plating (Supplemental Figure 7E). Regenerative capacity of *Bmal1^creERT2/+^* AT2 cells was furthermore reduced by 66% when cocultured with *Cry1,2*–DKO fibroblasts (Figure 3L; *P* = 0.0001, one-way ANOVA). Overall, our data are consistent with the circadian clock aiding lung regeneration via roles in both the alveolar epithelial and mesenchymal cells.

### Single-cell transcriptomic analysis of embryonic and postnatal KOs reveals differences in pathways involved in proliferation.

**Figure 4:**
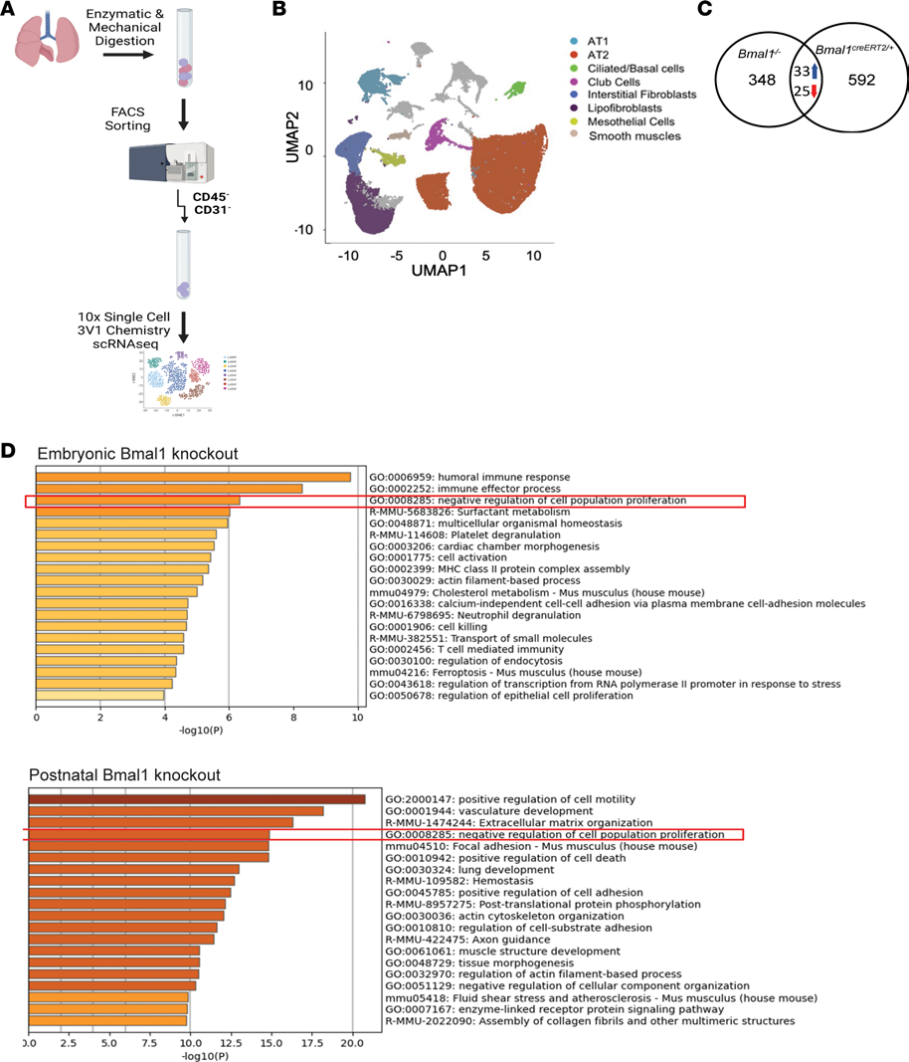
Single-cell transcriptome analysis of *Bmal1^–/–^* lung cells reveals downregulation of Wnt-associated pathways. (**A**) Schematic of experimental design for isolation of lung cells for single-cell sequencing from *Bmal1*^+/+^, *Bmal1*^–/–^, *Bmal1^creERT2/+^*, and *Bmal1^creERT2neg^* mice. DARQ7^–^CD31^–^CD45^–^ lung cells from uninfected mice underwent single-cell transcriptomic analysis. Data were integrated from *Bmal1^+/+^*, *Bmal1*^–/–^, *Bmal1^creERT2neg^*, and *Bmal1^creERT2/+^* mice. (**B**) Uniform manifold approximation and projection (UMAP) visualization dimension reduction analysis of integrated scRNA-seq data generated using a Seurat pipeline. (**C**) Venn diagram depicting the number of differentially expressed genes. (**D**) Enriched ontology clusters of differentially expressed genes in both the knockout models.

To elucidate the mechanistic basis of the regenerative deficiency seen with clock disruption, we undertook an unbiased approach with single-cell RNA sequencing (scRNA-seq) of epithelial and mesenchymal cells from *Bmal1*^–/–^, *Bmal1^creERT2/+^*, and their respective WT littermates (Figure 4A and Supplemental Figure 8A). A total of 52,580 CD45^–^CD31^–^ cells were sequenced based on our sorting strategy. Low-quality cells, defined as those that expressed fewer than 200 genes and/or genes greater than 2 median absolute deviations above the median, and with cells that had greater than 10% mitochondrial content were filtered out. Using the SCTransform function within Seurat (32), data were normalized, scaled, adjusted for percentage mitochondria, and number of unique molecular identifiers per cell, and the cell cycle phase score was calculated using the CellCycleScoring function in Seurat. The individual samples were integrated using normalized counts from SCTransform and the top 3000 variable genes as anchors. Principal component analysis (PCA) was used for linear dimension reduction, and subsequently ElbowPlot (https://satijalab.org/seurat/reference/elbowplot) was utilized for evaluation of the number of PCA dimensions. Data were clustered using the Louvain graph–based algorithm in Seurat at an appropriate cluster resolution. The uniform manifold approximation and projection (UMAP) dimension reduction algorithm was used to project the cells onto 2-dimensional space for visualization after integration of the postnatal and embryonic KO and WT data sets. The postnatal and embryonic KO and WT data sets were merged and projection of transcriptomic variations of individual cells by UMAP revealed 17 clusters, 10 of which were investigated. These were annotated based on the differential gene expression analysis of cell-type-specific known marker genes with highly different levels between clusters (33, 34) (https://research.cchmc.org/pbge/lunggens/) (Figure 4B and Supplemental Figure 8B). *Bmal1* deletion did not dramatically alter the overall clustering (Figure 4B); however, 406 genes in the embryonic KO model and 650 genes in the postnatal KO model were differentially expressed. Common to both models of *Bmal1* deletion, 33 genes were upregulated and 25 genes were downregulated in the KO versus WT (Figure 4C; source file 3, adjusted *P* value = 0.05, Wilcoxon’s method). We examined the top 20 Gene Ontology (GO) categories and discovered that genes involved in negative regulation of cell proliferation and lung development were highly enriched in both KO models (Figure 4D). Among genes involved in the regulation of cell proliferation, we found many that converged on the Wnt/β-catenin pathway (Supplemental Figure 8C). The Wnt/β-catenin pathway is critical for both lung development and lung repair and epithelial cell proliferation following lung injury (17, 31, 35). Activated canonical Wnt signaling has been shown to be involved in differentiation of lung epithelial stem/progenitor cells (17, 31, 36). Cyclin D promotes cell proliferation as a regulatory partner for CDK4 or CDK6 (37), and its homologs Ccnd1 and Ccnd3 were downregulated in *Bmal1^–/–^* lungs. Ccnd1 is a transcriptional target of the Wnt/β-catenin canonical pathway (38, 39). Another Wnt/β-catenin target gene, stearoyl-CoA desaturases2 (*Scd2*) (40), was upregulated in both KO models. Similarly, Cbp/p300-interacting transactivator with Glu/Asp-rich carboxy-terminal domain 2 (*Cited2*), a negative regulator of *Hif1a* whose gene product, Cbp/p300, interacts with β-catenin and is involved in the transcriptional regulation of Wnt signaling (41), was downregulated in both models of *Bmal1* deletion. *Lgals3* was one of the upregulated genes, and its product galectin-3 is a key regulator of the Wnt/β-catenin (42–44) signaling pathway through its interactions with Axin-2, GSK-3β, and β-catenin. Given the importance of the Wnt/β-catenin pathway in mediating lung proliferation and differentiation and its contribution to recovery from IAV, we hypothesized that clock-gated lung regeneration is mediated through Wnt-driven lung proliferation.

### Circadian disruption impairs cell proliferation in the lungs infected with IAV.

**Figure 5:**
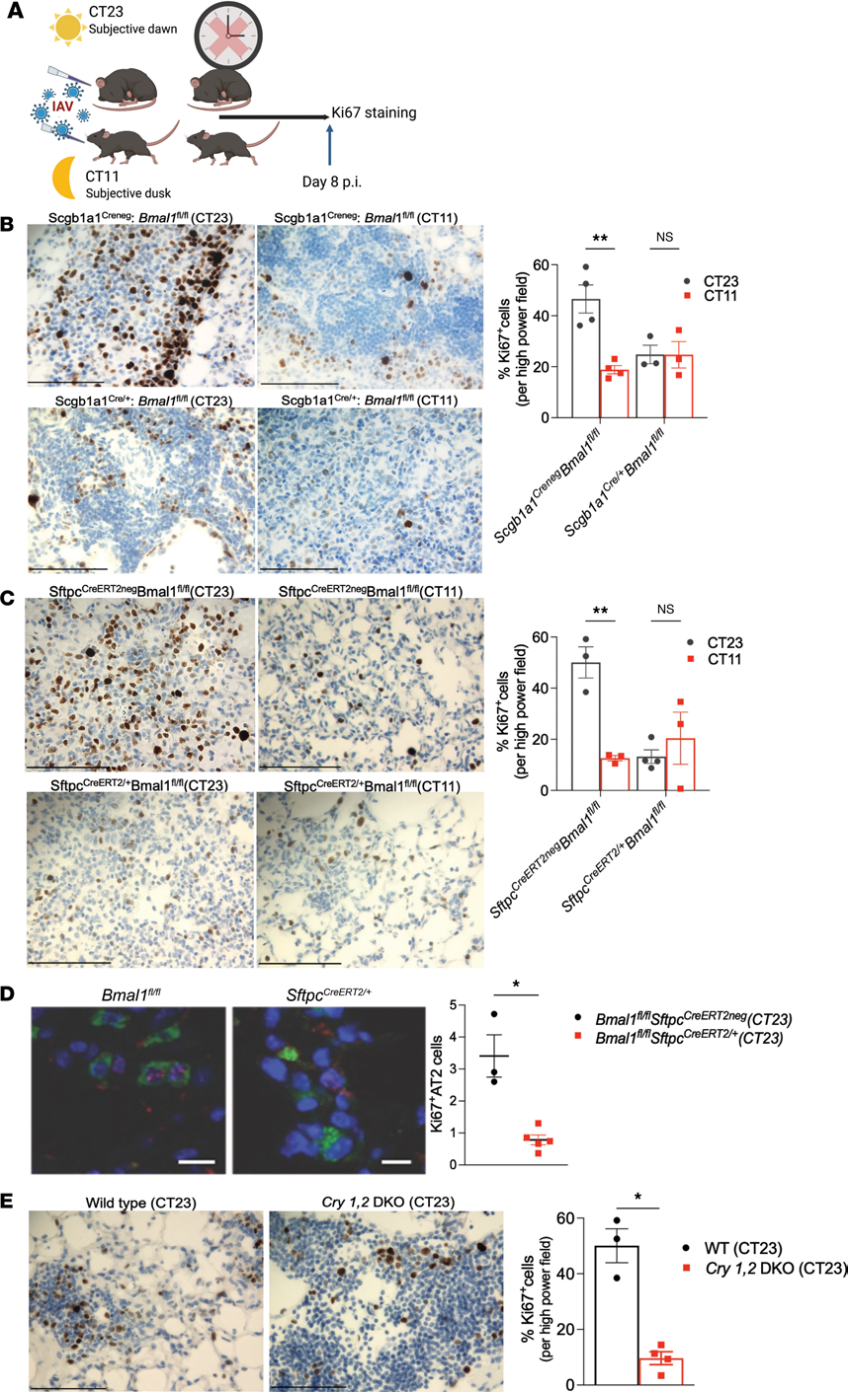
Disruption of the circadian clock decreases proliferation after IAV infection. (**A**) *Sftpc^CreERt2/+^ Bmal1^fl/fl^* mice, *Cry1,2*–DKO, and *Scgb1a1^Cre/+^ Bmal1^fl/fl^* and their cre^neg^ or WT littermates were moved to constant darkness (DD) 2 days prior to administering IAV at either CT23 or CT11. (**B**) Left: Representative Ki67-stained images from *Scgb1a1^Creneg^* *Bmal1^fl/fl^* mice and *Scgb1a1^Cre/+^* *Bmal1^fl/fl^* littermates. Right: Quantification of Ki67^+^ cells/high power field (HPF). *P* = 0.0939 for genotype, *P* = 0.0091 for time of infection, and *P* = 0.0094 for interaction by 2-way ANOVA; **P = 0.0012 for cre^neg^ group and *P* > 0.999 for cre^+^ group with Bonferroni’s correction for multiple comparisons. (**C**) Left: *Sftpc^CreERT2neg^ Bmal1^fl/fl^* mice versus *Sftpc^CreERt2/+^* *Bmal1^fl/fl^* littermates. Right: Quantification of Ki67^+^ cells/HPF. *P* = 0.0314 for genotype, *P* = 0.0265 for time of infection, and *P* = 0.0035 for interaction by 2-way ANOVA; **P = 0.0030 for cre^neg^ group and *P* = 0.75 for cre^+^ group with Bonferroni’s correction for multiple comparisons. (**D**) Images and quantification Ki67^+^ AT2 cells in *Sftpc^CreERT2neg^ Bmal1^fl/fl^* and *Sftpc^CreERt2/+^* *Bmal1^fl/fl^* mice. (**E**) *Cry1,2*–DKO mice and quantification. *P = 0.0129 by unpaired 2-tailed *t* test. Each point represents an animal and data are expressed as mean ± SEM. Scale bars: 100 μm.

To test whether disruption of the circadian clock impaired proliferation following IAV infection, based on existing literature and our own work (Supplemental Figure 9A), we selected day 8 p.i. to be an ideal time point for evaluation of lung proliferation. First, we infected *Scgb1a1^Cre/+^ Bmal1^fl/fl^* and their cre^neg^ littermates with IAV at either CT11 or CT23 and harvested lungs on day 8 p.i.(Figure 5A). We found that both cre^+^ groups (infected at CT23 or CT11) had fewer Ki67^+^ cells per high-power field (HPF) and thus lower proliferation than the *Scgb1a1^Creneg^* *Bmal1^fl/fl^* mice infected at CT23 (Figure 5B). However, the temporal difference in cell proliferation was still maintained in *Scgb1a1^Creneg^* *Bmal1^fl/fl^* mice, where mice infected at CT23 had greater than 2-fold higher proliferation than those infected at C11 (Figure 5B). However, proliferation of AT2 cells is also needed for optimal lung repair. Next, to investigate whether the disruption of the AT2 clock impaired proliferation following flu, we infected *Sftpc^Cre-ERT2/+^ Bmal1^fl/fl^* (in which *Bmal1* was selectively deleted in AT2 cells; Supplemental Figure 9B) and their cre^neg^ littermates at either CT11 or CT23. Fewer proliferating or Ki67^+^ cells/HPF were observed in animals infected at CT11 versus CT23 (Figure 5C; 12% at CT11 vs. 50% at CT23, *P* = 0.005) in *Sftpc^CreERT2neg^* *Bmal1^fl/fl^* mice. This time-of-day–specific protection was lost in *Sftpc^Cre-ERT2/+^ Bmal1^fl/fl^* littermates, wherein both groups had lower and comparable Ki67^+^ cells/HPF (cre^+^ 11.73% in CT23 and 20.42% in CT11 vs. 50% among cre^neg^ CT23; *P* = 0.01), suggesting that disruption of the AT2 clock by deletion of *Bmal1* impaired proliferation that is necessary for optimal recovery from IAV (Figure 5C). Specifically, we confirmed that there were fewer Sftpc^+^Ki67^+^ cells in the *Sftpc^Cre-ERT2/+^ Bmal1^fl/fl^* than their cre^neg^ littermates (Figure 5D). To further investigate the effect of clock disruption on epithelial proliferation, we also performed organotypic assays after infection. While WT mice had a robust increase in their regenerative capacity after infection (AT2 cells harvest on day 8 p.i.), *Bmal1^creERT2/+^* AT2 cells failed to show any appreciable increase in their regenerative capacity (Supplemental Figure 9C). Finally, to confirm that the impaired proliferation seen above was not secondary to noncircadian roles of *Bmal1*, we repeated the experiment with *Cry1,2*–DKO mice. We found that there were very few Ki67^+^ cells/HPF in the *Cry1,2*–DKO mice compared with WT controls on day 8 p.i.(Figure 5E; 9.6 % in *Cry1,2*–DKO mice vs. 50% in WT cells; *P* = 0.001). Thus, the circadian clock modulates regenerative capacity by impairing proliferation in the aftermath of IAV.

### Bmal1 directly binds to E-box motif in the Wnt3a promoter.

**Figure 6:**
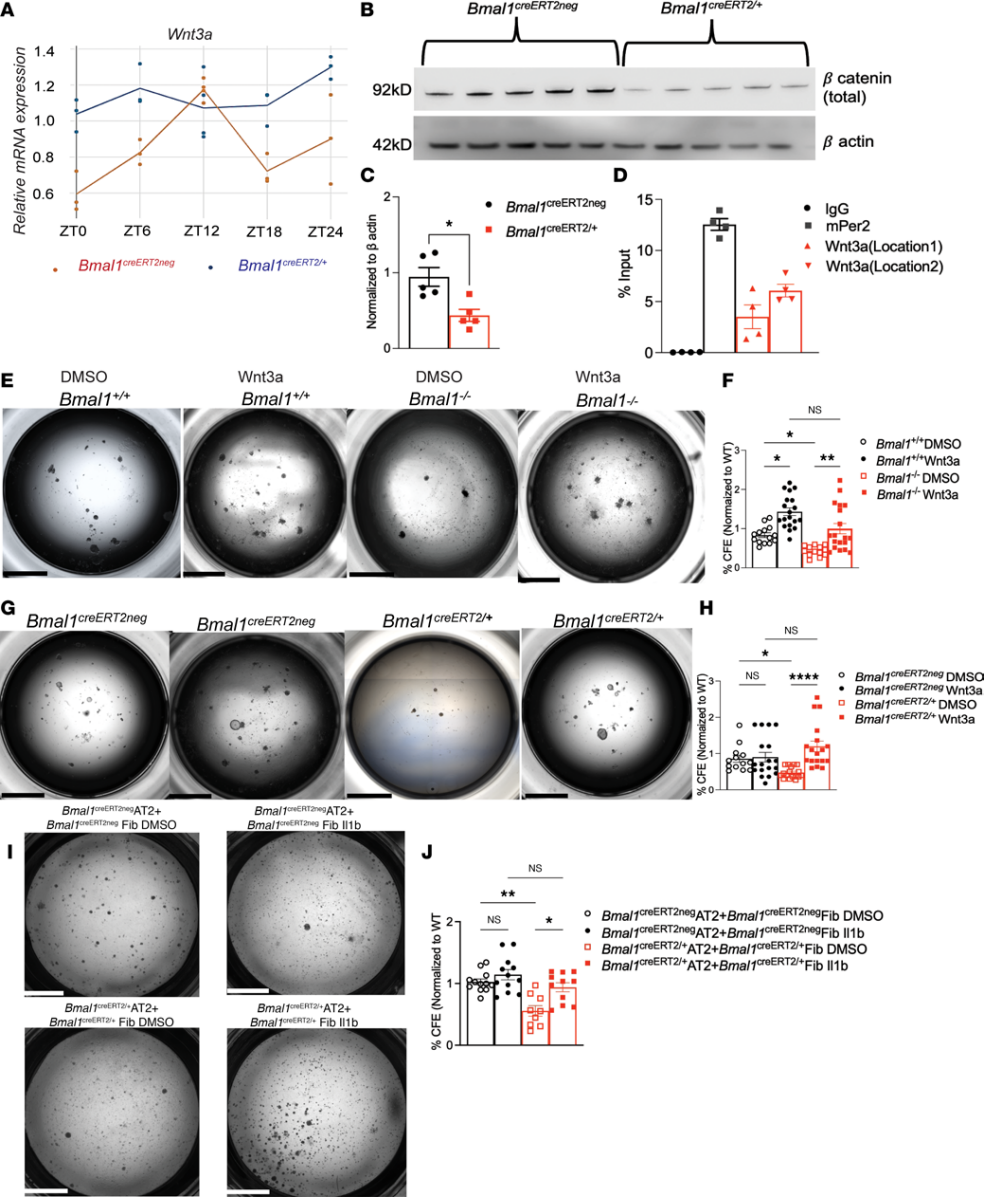
Activation of Wnt signaling in tracheal organoids rescues the regenerative defect in the absence of *Bmal1*. (**A**) Gene expression of *Wnt3a* from *Bmal1^creERT2neg^* lungs harvested at different time intervals determined by qPCR (*n* = 3–4 mice per time point, *q* = 0.014). (**B**) Representative immunoblot of β-catenin expression from whole-lung extracts from *Bmal1^creERT2neg^* and *Bmal1^creERT2/+^* mice. (**C**) Quantification of β-catenin expression normalized to β-actin from 2 independent experiments (*n* = 4–5, *P < 0.01). (**D**) ChIP assay of BMAL1 occupancy on the *Wnt3a* promoter. *mPer2* primers were used as positive controls for the analysis. Data are expressed as percentage of input level normalized to IgG control (*n* = 4, pooled from 2 independent experiments). *P = 0.04, unpaired 2-tailed *t* test with Welch’s correction. Tracheal organoids were supplemented with Wnt3a in DMSO. (**E**) *Bmal1^+/+^* WT littermates, *Bmal1^–/–^* DMSO, *Bmal1^–/–^* Wnt3a. (**G**) *Bmal1^creERT2neg^* littermates DMSO, *Bmal1^creERT2/+^* DMSO, and *Bmal1^creERT2/+^* Wnt3a CFE for (**F**) *Bmal1^–/–^* and (**H**) *Bmal1^creERT2/+^*. *P = 0.01,**P = 0.001, ****P = 0.0001. (**I**) AT2 organoids were supplemented with IL-1β (10 ng/mL) and DMSO (0.05% final concentration). (**J**) CFE: *P = 0.04, **P = 0.008, ***P = 0.0004. Scale bars: 2000 μm. Data pooled from 3–5 independent experiments with at least 3 technical replicates/experiment expressed as mean ± SEM. Kruskal-Wallis test with Dunn’s multiple-comparison test.

To test whether the Wnt/β-catenin pathway is under clock control and validate our findings from the scRNA-seq experiment, we harvested lungs at 6-hour intervals and checked for gene expression (Figure 6A and Supplemental Figure 10A). We were able to thus confirm that not only does Wnt3a show circadian rhythmicity (*q* value = 0.014 by JTK), but also that this rhythmicity is lost when the clock has been disrupted by *Bmal1* deletion in *Bmal1^fl/fl^* ERT2-cre^+^ mice (Figure 6A). This rhythmicity is lost in the *Bmal1*-null (*Bmal1^fl/fl^* ERT2-cre^+^) mice, which we posit suggests the lack of circadian control over Wnt3a regulation and by extension on proliferation. Canonical Wnt signaling involves β-catenin stabilization and accumulation in the cytosol. We determined β-catenin abundance in the lungs as a measure of Wnt activity in naive lungs from both *Bmal1^creERT/+^* mice as well as cre^neg^ littermates. Cytosolic β-catenin expression was significantly lower in the lung protein extracts of the *Bmal1^creERT2/+^* than in their *Bmal1^creERT2neg^* littermates, suggesting that BMAL1 may directly target the Wnt pathway (Figure 6, B and C). Supporting this, other downstream targets of Wnt3 showed remarkable circadian rhythmicity in the lung (Supplemental Figure 10B). In turn, this downregulation at baseline could result in impaired activation of downstream Wnt signaling and account for the loss of regenerative capacity seen in our models of clock disruption.

Therefore, we further investigated whether Wnt3a is a direct transcriptional target of BMAL1 using chromatin immunoprecipitation (ChIP). Using bioinformatic analyses, we found that the promoter region of *Wnt3a* contained 3 potential E-box motifs (CACGTG) to which BMAL1 could be recruited. We then compared the fold enrichment of Wnt3a and Per2 (a known target of BMAL1 protein) following ChIP with anti-BMAL1 antibodies. *Per2* showed approximately 12.5-fold enrichment of BMAL1 recruitment to its promoter over IgG control (Figure 6D). Wnt3a was 6.12-fold enriched over its control, confirming that BMAL1 protein binds to the E-box motif in the *Wnt3a* promoter (Figure 6D). Thus, we show that Wnt3a can serve as a direct target of the circadian clock via BMAL1. Thus, we next test whether activation of the Wnt3a signaling pathway can rescue the phenotype associated with *Bmal1* deficiency–related clock disruption.

### Wnt3a treatment rescues Bmal1^–/–^ phenotype in tracheal organoids.

Next, we investigated whether activating Wnt signaling in the tracheal organoids with a disrupted clock would be sufficient to rescue the regenerative deficiency noted earlier. Supplementing *Bmal1^–/–^* tracheal cells with Wnt3a resulted in a 2.2-fold increase in its regenerative capacity, making it comparable to organoids grown from *Bmal1*-sufficient cells in standard media (Figure 6, E and F; *P* = 0.01, Kruskal-Wallis test). Similar results were noted in the *Bmal1^creERT2/+^* model in which the regenerative deficiency was also comparably rescued with Wnt3a supplementation (Figure 6, G and H; *P* = 0.01, *P* = 0.0001, Kruskal-Wallis test).

This contrasted with alveolar organoids, in which supplementation with Wnt3a or the Wnt pathway activator CHIR (see Supplemental Methods) did not rescue the regenerative loss associated with *Bmal1* deficiency, suggesting the role of additional circadian-controlled mechanisms in AT2 regeneration (Supplemental Figure 10, D–F). Given the role of inflammation in lung repair and regeneration (45, 46), as well as our data consistent with lingering effects of inflammation in our models, we considered inflammatory mediators that affect lung regeneration and are known to be under circadian control, such as IL-1β, which enhances regenerative responses in the lung but is also known to be downstream of clock-gated inflammation (47). Interestingly, we have previously demonstrated that IL-1β expression was suppressed in *Sftpc^Cre-ERT2/+^ Bmal1^fl/fl^* mice compared with their cre^neg^ littermates (12). Thus, we next supplemented AT2 organoids in the above experimental setup with IL-1β and observed that this was in fact able to rescue the regenerative deficiency due to disruption of the AT2 clock (Figure 6, I and J, and Supplemental Figure 10F). Taken together, these results show that clock disruption affects lung regeneration in different parts of the lung. However, the mechanisms through which this control is exerted are distinct and specific to the epithelial subtype.

## Discussion

Here, we studied the effect of circadian rhythms on long-term outcomes following viral infection through the processes of lung repair and regeneration. We used valuable epidemiological insights from the UK Biobank to drive our mechanistic studies, employing a wide range of models of circadian disruption.

We found that poor circadian rhythms were associated with increased likelihood of hospitalization for respiratory infection, longer duration of hospital stay, and increased mortality both in hospital but also 30 days after discharge. While these analyses were not designed to unequivocally prove causality, we find the consistent association between less robust circadian rhythms and several measures of poor outcomes from lower respiratory tract infections a compelling argument for further study of this risk factor.

Our group (6, 12, 48) as well as others (5, 48) have shown that circadian control of acute mortality and morbidity is manifested in a time-of-day–specific protection from the ensuing inflammation. Here, we report that the circadian influence persists well beyond the acute phase, with several measures of poor repair and regeneration observed in the group infected at dusk. We found that circadian rhythms regulate lung proliferation, repair, and regeneration independently of clock-gated lung inflammation. Differences in peak inflammation, cell death patterns, delayed resolution, changes in immune subsets that exert a predominantly antiinflammatory effect, and other repair pathways can also affect the final outcomes as seen both in our histology from the murine model and the UK Biobank data. While detailed analyses of all these factors is beyond the scope of the current paper, future studies investigating these effects will be critical for harnessing circadian mechanisms to improve lung health.

Interestingly, the directionality of this protection is the opposite of what is observed for wound healing in the skin, in which healing was better for wounds sustained at night (8), suggesting that circadian regulation is unique to the needs of each organ. We demonstrated that epithelial cells from different levels of the respiratory tract with facultative stem cell function have a functional intrinsic clock and disruption of this circadian clock leads to loss of their regenerative potential. We compared the lung transcriptome at the single-cell level of both embryonic and postnatal *Bmal1*-KO models to dissect the signatures associated with loss of the circadian clock. Although there were many implications of cell proliferation and differentiation from the differential gene expression profile, overall, the result was subtle. However, this differential expression is likely an underestimation of the true functional effect of the clock following injury since the epithelium in an uninjured lung is rather quiescent and not actively proliferating. On the other hand, the use of uninfected lungs allowed us to circumvent signals emanating from clock-gated regulation of the inflammation induced by IAV.

While the connection between the circadian clock and the Wnt/β-catenin pathway has not been investigated before in the lung to the best of our knowledge, *Wnt* activation in adipose tissue was suppressed specifically in *Bmal1^–/–^* mice, which in turn impaired adipogenesis (49). Another study has demonstrated that the circadian clock synchronizes the timing of cell division via rhythmic secretion of Wnt3a in intestinal organoids (9). Activated canonical Wnt signaling has been shown to be crucial for proliferation, survival, and differentiation of lung epithelial stem/progenitor cells (17, 31, 36). Based on these clues from the literature and our results from the scRNA-seq and lung proliferation, we focused on the Wnt/β-catenin signaling pathway. This was validated by the expression of the *Wnt3a* gene, which showed rhythmic expression in WT mice, while this rhythmicity was lost in clock-disrupted mice. We also found that upon disruption of the clock with *Bmal1* deletion, β-catenin was downregulated. Furthermore, in tracheal organoids, the deficiency of regenerative capacity seen with *Bmal1* deletion could be completely rescued by exogenous administration of Wnt3a. Overall, these data confirm circadian control over Wnt-driven lung regeneration.

While there was loss of regenerative capacity in AT2 organoids from different models of clock disruption, the phenotype suggested a role for both the epithelium and mesenchyme that was rescued not by Wnt3a, but rather by IL-1β supplementation. However, the in vivo data clearly showed a loss of proliferative response in the absence of an intact AT2 clock. Considered together, this would suggest that both inflammatory and inflammation-independent circadian pathways control alveolar repair and regeneration following IAV. Interestingly, while IL-1β rescued the regenerative deficiency of AT2 organoids grown from *Bmal1*-deficient animals, in peritoneal macrophages, *Bmal1* deficiency enhances IL-1β expression (47, 50). Thus, our data support the finding that the circadian-controlled targets and processes are unique to each tissue type and condition.

Future studies will be needed to elucidate the exact mechanism(s) underlying the coupling of inflammation and lung repair and regeneration in AT2 cells. The role of clock-gated interactions between the mesenchymal and epithelial compartments will also be important for a complete understanding of circadian regulation of lung repair and regeneration, as suggested by our organoid assays. Overall, the consistent and unambiguous phenotype of poor lung proliferation resulting in poor repair and regeneration following IAV across models of clock disruption underscore the importance of our work. Our studies are strengthened by a combination of models for circadian disruption, including the use of both postnatal and embryonic *Bmal1^–/–^* and *Cry1,2*–DKO models, which allows us to differentiate the circadian versus noncircadian roles of BMAL1.

In summary, through our current work, we introduce a circadian dimension to the complex process of lung repair and regeneration. The state of circadian health of an individual may be an important predictor of how one recovers following lung injury. This may have several implications for patients suffering from lung injury of various etiologies, well beyond IAV. To the best of our knowledge, this is the first study linking the circadian clock with lung repair and regeneration. Consideration of circadian context of lung injury and repair may not only provide novel therapeutic strategies but also allow for improved efficacy of many of our existing therapies toward enhancing lung recovery.

## Methods

Further details can be found in the Supplemental Methods.

### UK BioBank analyses.

Actigraphy data were collected on 103,685 patients in the UK Biobank (https://www.ukbiobank.ac.uk/) for 1-week recordings. The data were calibrated and processed using an existing pipeline (16).

### Lung injury.

Mice were intranasally infected at either ZT11 (1 hour before onset of activity phase or dusk) or ZT23 (1 hour before onset of rest phase or dawn) with IAV (PR8). By circadian nomenclature, ZT0 refers to time at which lights went on. To remove any confounding effect of light-driven behavioral rhythms, all transgenic mice were infected under constant darkness. The times corresponding to ZT11 and ZT23 in constant darkness are referred to as CT11 (subjective dusk) and CT23 (subjective dawn) respectively, by circadian convention.

### Data availability.

The scRNA-seq data are available in the NCBI Gene Expression Omnibus (GEO GSE189500). All other data are available as source data.

### Study approval.

All animal studies were approved by the University of Pennsylvania Animal Care and Use Committee (protocol ID 805645) or the Children’s Hospital of Philadelphia (protocol ID 21-001405) and met the stipulations of the NIH *Guide for the Care and Use of Laboratory Animals* (National Academies Press, 2011). UK Biobank data were obtained following standard protocols. Data are available as a deidentified database that included information on specific query items.

## Author contributions

SS conceived the project. AN, SS, EEM, GSW, and AS designed experiments. AN, KMF, YI, UKV, DBF, SYT, JAZ, and SS performed experiments and collected data. OP designed and performed experiments. FC and EJH performed experiments. AN, AB, MM, and TGB analyzed data. ABR and DBF were involved in the interpretation of data. GAF, GRG, and TGB were involved in the UK Biobank analyses. AS also provided *Cry1,2*–DKO mice. AN wrote original draft of the manuscript with help from SS. SS supervised all research activities.

## Supplementary Material

undefined

## Version 1 07/18/2023

In-Press Preview

## Version 2 08/22/2023

Electronic publication
