# Supplementary material for: Circadian regulation of lung repair and regeneration
Source: JCI Insight. 2023 Aug 22;8(16):e164720. doi: 10.1172/jci.insight.164720 (PMC10543710; doi:10.1172/jci.insight.164720)
Supplement: undefined [file jciinsight-8-164720-s094.pdf]

Supplemental Methods:

UK BioBank analyses (continued):

Models were run using the survival version 3.2-13 package with R. Results are reported as hazard ratios comparing top or 5th quintile RA (highest or best circadian rhythms) to bottom or 1st quintile RA (lowest or worst circadian rhythms) with a robust 95% confidence interval. All models were assessed for meeting the assumptions of Cox proportional hazards models. Shoenfeld residuals were plotted versus time and checked with the cox.zph function. Circadian rhythms were measured as the relative amplitude (RA) defined as (M10 – L5)/(M10 + L5), where M10 and L5 are the average acceleration levels during the highest 10 hours of activity and the lowest five hours of activity. Overall physical activity (PA) levels were measured by the average acceleration. Of these, 84,823 subjects had valid actigraphy data. Pneumonia, influenza, and acute lower respiratory infections were identified from in-patient hospital record ICD-10s codes J09-J22, for a total of 4056 hospital admissions involving these diagnoses across 3493 patients. 94% of the diagnoses were listed as “J22-Unspecificed acute lower respiratory infection” for this cohort. Cox proportional hazards models were run for each of hospital admission by RA score quintile. These were repeated with outcome as death during hospitalization and as death within 30 days of discharge. All these models used natural age as the time scale and included as covariates age at the time of actigraphy measurement, physical activity quintile, sex, BMI, prior history of pneumonia or flu hospitalization, lifetime smoking history (ever/never), self-reported health, and use of certain medications pertaining to respiratory diagnoses (inhaled steroids and/or bronchodilators). Lastly, hospital stay duration was modeled by RA score quintile with the same set of covariates. Days since admission was the timescale and death was treated as a competing outcome.

Mice.

Embryonic *Bmal1* knockout mice ($Bmal1^{-/-}$)(1) where deletion of *Bmal1* prenatally disrupts clock-dependent oscillatory gene expression and behavioral rhythmicity and their littermate controls ($Bmal1^{+/+}$) were generated by in-house breeding. $Bmal1^{creERT2/+}$ mice were purchased from Jackson labs (B6.Cg-Tg(CAG-cre/Esr1*)5Amc/J Strain # 004682) and postnatal global $Bmal1^{-/-}$ mice were generated as described previously(1). AT2 cell-specific knockout of *Bmal1* ($Sftpc^{Cre\text{-}ERT2/+}$:$Bmal1^{fl/fl}$) was generated by crossing $Sftpc^{Cre\text{-}ERT2/+}$, a tamoxifen-inducible Cre, with $Bmal1^{fl/fl}$ mice(2). To generate postnatal *Bmal1* knockout mice, 2 months old (unless specified) $Bmal1^{creERT2/+}$ mice were treated with 5mg (in 50µl) of tamoxifen via oral

gavage, for 5 consecutive days. Tamoxifen was reconstituted to 100mg/ml solution with ethanol and corn oil and thawed at 55°C prior to administration. *Bmal1*^{creERT2neg}, *Sftpc*^{CreERT2neg}*: Bmal1*$^{fl/fl}$ littermates treated with tamoxifen served as controls. A 2-week washout period was used for all experiments. Club cell specific *Bmal1 knockout Cite (Scgb1a1*$^{Cre/+}$*: Bmal1*$^{fl/fl(3)}$*) was* generated by crossing *Scgb1a1-cre*$^{+}$*,* with *Bmal1*$^{fl/fl}$ mice, *Scgb1a1*^{Creneg}*:Bmal1*$^{fl/fl}$ served as controls(4) [S Fig 9D]. PER2::LUC mice (B6.129S6-Per2^{tm1Jt}/J), in which the luciferase gene is fused in-frame to the 3′ end of the endogenous mPer2 gene (one of the core clock genes), were purchased from Jackson Laboratory animal facility (stock: 006852). For luciferase assay, tracheal cells, CD104^{+} distal lung cells or AT2s were harvested from *these mice*. *Cry1*$^{-/-}$*Cry2*$^{-/-}$ *(Cry1,2 DKO)* mice were a gift from Dr. Amita Sehgal, HHMI, University of Pennsylvania, Philadelphia(5, 6). Male and female mice were used in approximately equal proportion for all experiments. In all experiments with clock mutant models, WT littermates were used as controls. Unless specified otherwise tissues were harvested at ZT1-2.

Lung Injury

For influenza infections, mice were lightly anesthetized with isoflurane and infected intranasally (i.n.) with sub-lethal dose of IAV (H1N1:PR8,sub-lethal dose), in a volume of 40 μl at the times and under light-dark conditions as indicated in the specific experimental strategy.

Organoid assays

General information for organoid assays: Only two animals (KO vs control) were sorted at each time to ensure that the general health of the cells was optimized. All harvests were done between ZT1-3, based on availability of sorter in the Flowcytometry Core.

Tracheal organoids: Mice trachea were processed by peeling off the epithelium and digesting in a 1:1 mixture of DNAse II (Roche) and Liberase (Roche) for 30 min at 37°C. After centrifugation at 1200 rpm, the cell pellet was re-suspended in 0.25% trypsin EDTA and incubated at 37 °C for 10 min. Thereafter the cell suspension was filtered through a 40 μm cell strainer (BD Biosciences) and recovered cell pellet was plated in the appropriate media. Tracheal cells (60,000-100,000 cells/ well in 96 well plate) were cultured in small airway growth media (SABM™ Basal Medium, CC-3119 Lonza) containing 1× insulin, 1x transferrin,1x EGF, 1x Retinoic acid, and 1x bovine pituitary extract (SAGM™ SingleQuots™ supplements, CC-4124 Lonza), 0.1 μg/ml Cholera Toxin (C9963 Sigma), and 5% FBS (Denville) on growth factor–reduced, phenol-free matrigel (356231 Corning).

Bronchial epithelial organoids and AT2 organoids: Lungs were harvested after PBS perfusion followed by digestion with DNAse II and Liberase at 37°C for 20 mins. Dissociated lung tissue

was passed through a 70 µm cell strainer (BD Biosciences), followed by centrifugation and RBC lysis. After magnetic bead-based depletion of leukocytes and endothelial cells ($CD45^-CD31^-$), the single cell suspension was sorted for alveolar epithelial cells (AT2 as $DRAQ7^-$ $EPCAM^+CD104^-$) and $CD104^+$bronchial epithelial cells ($DRAQ7^-EPCAM^+CD104^+$) on BD FACSJazz™ (S. Fig 2A). Using this strategy, the alveolar fraction, is likely to contain some AT1 along with AT2 cells and the CD104+ fraction has a mix of bronchial epithelial cells. Epcam+ $CD104^-$ population was used as a surrogate for AT2 organoids. This was confirmed on histology. Similarly, the $CD104^+$ organoids, represented the regenerative capacity of a mix of basal and club cells.

Lung fibroblasts for organoid assays were isolated from embryonic *Bmal1*$^{-/-}$, wild type littermate *Bmal1*$^{+/+}$ controls, *Bmal1*$^{creERT2/+}$, and *Bmal1*^{creERT2neg} littermate controls. To do so, lungs were harvested, digested, and processed into a single cell suspension as described above. Thereafter they were plated and serially passaged in DMEM/F12 (11320033 Life Technologies) supplemented with 10% FBS and penicillin-streptomycin (P433310,000 U/mL, Sigma) 3 times.

15,000-30,000 cells/well of $CD104^+$basal cells sorted from distal lung, were cultured in C12 media supplanted with growth factors onto growth factor–reduced, phenol-free matrigel depending on the yield of cells per experiment per experimental condition in 96 well plate.

<u>For alveolar organoid assays</u>: For each experiment, 5×10^3 $Epcam^+CD104^-$ were isolated as described above and mixed with 5×10^4 lung fibroblasts. Cells were then suspended media similar to tracheal organoids and growth factor-reduced, phenol-free matrigel at 1:1 concertation. 90µl of the cell/media/matrigel mixture was then aliquoted into individual 24-well cell culture inserts and allowed to solidify at 37^0C. Complete SAGM was added to each well. Rock inhibitor (Y27632 Sigma) was included in the media for the first two days. Cultures were maintained at 37^0C, 5% CO2. Media was replenished every 48h until day 8 for tracheal and CD104+organoids, and day 21 for AT2 organoids as described previously(7, 8).

Real-time bioluminescence recording of PER2::LUC organoids

For luciferase assay, tracheal cells, $CD104^+$ distal lung cells or AT2s were harvested from *mPer2*^{luc} mice (Stock No: 006852). Bioluminescence was recorded without any synchronization agents. Bioluminescence outputs were recorded by adding beetle luciferin potassium salt (E1602 Promega) to organoid media at a final concentration of 1 mM. Cultures were

monitored for light output using a custom-made bioluminescence recording system (Cairn Research Ltd, UK) composed of charge-coupled device camera (Andor iKon-M 934) mounted on the top of an Eppendorf Galaxy 170R CO2 incubator. Since the tracheal and CD104+ organoids mature by day 7 and AT2 organoids mature by day 21, they were placed in the bioluminescence chamber on days 4 and 18 respectively. Background was subtracted and bioluminescence data traces were analyzed with a modified R script CellulaRhythm(9).

Flowcytometry:

As described previously, lungs were perfused with PBS through the right ventricle and digested using DNAse II (Roche) and Liberase (Roche) at 37°C for 30 mins. The left lung was harvested and dissociated tissue was passed through a 70 μm cell strainer, followed by centrifugation and RBC lysis. Cells were washed and re-suspended in PBS with 2% FBS. 2-3 x 10^6 cells were blocked with anti-CD16/32 antibody and stained with indicated antibodies on ice for 20-30 minutes. No fixatives were used. Flowcytometric data was acquired using FACS Canto flow cytometer and analyzed using FlowJo software (Tree Star, Inc.). All cells were pre-gated on size as singlet, live cells. All subsequent gating was on CD45$^+$ in lung only. Neutrophils were defined as CD45$^+$Ly6G$^+$, inflammatory monocytes as CD45$^+$CD11b$^+$Ly6C^{hi}Ly6G$^-$ cells and virus specific T cells as PA-224$^+$CD62L^{lo}CD44$^+$CD8$^+$CD45$^+$ cells (kind gift from John Wherry, University of Pennsylvania, Philadelphia originally obtained from NIH tetramer facility).

H & E staining scoring: Lung injury post 30 days IAV infection was assessed by H&E staining. We used Image J to overlay a grid on the whole lung cross section and allocated each square contained in the grid into one of four zones based on the severity of the injury as defined previously; Zone 1-minimal injury, Zone 2-minor injury with mild interstitial thickening, Zone 3-severe alveolar injury or Zone 4- complete alveolar destruction(10).

Single cell preparation, and sorting

Lungs from embryonic *Bmal1*$^{-/-}$, *Bmal1*$^{+/+}$ controls, *Bmal1*$^{creERT2/+}$, and *Bmal1*^{creERT2neg} littermates (n=1 per genotype) were dissected, and single-cell preparation was obtained as described above (under organoid assay). Single cell suspension was sorted as DRAQ7$^-$CD31$^-$CD45$^-$ lung cells [Suppl Fig 4A] The sorted cells were loaded onto a Chromium Controller instrument (10× Genomics, Pleasanton, CA, USA) to generate single-cell barcoded droplets (GEMs) according to the manufacture's protocol using the 10× Single Cell 3' v1 chemistry. The resulting libraries were uniquely indexed using the Chromium i7 Sample Index Kit, pooled, and sequenced were sequenced on the Illumina NovaSeq 6000 sequencer using an SP 100 cycles flow cell in a

paired-end, single indexing run. Sequencing for each library targeted 25,000 mean reads per cell. Data was then processed using the Cell ranger pipeline (10x genomics, v.3.1.0) for demultiplexing and alignment of sequencing reads to the mouse mm10 transcriptome and creation of feature-barcode matrices. Individual single cell RNAseq libraries were aggregated using the cell ranger aggr pipeline. Libraries were normalized for sequencing depth across all libraries during aggregation. Seurat and Loup browser were used for further processing and downstream analysis(11). Differentially expressed genes from both *Bmal1* models were analyzed for enriched ontology clusters on mescape.org.

Chromatin Immunoprecipitation

Lungs were cut into small pieces and cross-linked with 1% formaldehyde for 10 min at room temperature (RT). 125mM of glycine (Thermo Fisher Scientific) was added to stop the crosslinking reaction with additional incubation for 5 minutes on the revolver at RT. Tissue was homogenized for 10s at 06m/sec on MP Fastprep-24 5G (MP Biomedicals). Cell pellet was washed with cold PBS, centrifuged, and then suspended in swelling buffer (5 mM PIPES pH 8.0, 85 mM KCl, 1% NP40, and protease inhibitor cocktail) for 30 minutes on ice. The crude nuclear preparation was centrifuged, and nuclei were suspended in nuclear lysis buffer (50 mM Tris-HCl pH 8.0, 10 mM EDTA, 1% SDS, and protease inhibitor cocktail) and sonicated to an average length of about 300-500 base pairs using Diagenode Bioruptor (UCD-200), at 4°C on setting of “3” for 35 minutes.

Sonicated samples were diluted 10-fold with IP dilution buffer (16.7 mM Tris-HCl pH 8.0, 0.01% SDS, 1.1% Triton X-100, 1.2 mM EDTA, 167 mM NaCl, and protease inhibitor cocktail) and incubated with anti-Bmal1 antibody (5 µg, ab3350, abcam) overnight at 4°C. DNA complexes were collected on Dynabeads protein A® (1001D, Thermofisher) and serially washed once with dialysis buffer (50 mM Tris-HCl pH 8.0, 2 mM EDTA, and 0.2% sarkosyl), three times with IP wash buffer (100 mM Tris-HCl pH 9.0, 500 mM LiCl, 1% NP40, 1% Deoxycholate, and protease inhibitor cocktail), and finally in TE buffer (1M TrisCL, 05.M EDTA, pH 8.0). Samples were eluted with elution buffer (50 mM $NaHCO_3$ and 1% SDS). Crosslinking was reversed by overnight incubation with 0.3 M NaCl at 65°C followed by proteinase K treatment (0.5 M EDTA, 1 M Tris-HCl (pH 7.5), and 10 mg/mL proteinase K) and DNA clean up (MiniElute reaction clean up kit, 28204, Qiagen). qPCR was performed using mPer2 and Wnt3a primers [Source file 3]. Rabbit IgG antibody (CS200581, Millipore) was used as a negative control and anti-Histone H3 (tri methyl K9, AB6002, Abcam) antibody was used as a positive control. Data was analyzed using % Input method.

Bronchoalveolar Lavage: Flu infected and control mice were euthanized by CO_2 asphyxiation on day 8 post infection, and their tracheas cannulated with a 20 G flexible catheter (Surflo, Terumo, Philippines). The lungs were gently lavaged with 600 µl of PBS in four passes. The supernatant from the first pass was collected for BAL total protein analysis (BCA Assay; Pierce Biotechnology, Rockford, IL, USA.

Quantification of lung collagen content. Total right lung acid soluble collagen content was determined using the Sircol assay (Biocolor Ltd.) according to the manufacturer's instructions.

Ligand treatments of tracheal and AT2 organoids were performed using the following reagents at the indicated concentrations: Wnt3a 200ng/ml (315-20 Peprotech), Fgf10 50ng/ml (100-26 Peprotech), CHIR99021 3 µm/ml (72052 StemCell Technologies), Recombinant mouse IL-1b 10ng/ml (575104 Biolegend), and DMSO (final concentration 0.05%, D2650 Sigma) was used a control. Each organoid assay was run with at least three technical replicates. Images were recorded using EVOS M7000 imaging system (Thermo Fisher Scientific). Colony forming efficiency (CFE: number of colonies formed/number of cells plated/well) of organoids was determined using a customized macro in Image J 1.47.

Histology (lung and organoids)

Lungs harvested at the end of the experiment were fixed by inflation with 10% buffered formalin at 20 mm H_2O of pressure, paraffin embedded, and stained with H&E stain. Stained slides were digitally scanned at 40x magnification using an Aperio CS-O slide scanner (Leica Biosystems, Chicago IL). Representative images were taken from scanned slides using Aperio ImageScope v12.2.2.5015 (Leica Biosystems, Chicago, IL). Three-four random fields per lung section were imaged for Ki67 scoring.

Similarly, after 8 days for tracheal and $CD104^+$, and 21 days for AT2 organoids were fixed in 2% paraformaldehyde, embedded in Histogel (Richard-Allen), dehydrated, paraffin embedded, and sectioned. Lungs and organoid were stained with hematoxylin and eosin to examine histology and organoid morphology respectively. Day 30 lungs were stained with CD3 (1:200, goat, sc-1127 Santa Cruz) and F4/80 antibody (1:200, rat, eBiosciences 14-4801). The images were analyzed using Aperio ImageScope (v12.4.3.5008). Immunofluorescence was performed following heat antigen retrieval methods and stained with the following antibodies. SFTPC (1:100, anti Pro Surfactant protein, rabbit, AB3786 Millipore), Ki67 (1:200, mouse, 550609 BD

Biosciences), Scgb3a2 (1:50 anti-Mugrp1, goat, AF3465 R&D systems), KRT5 (1:100, rabbit, 905504 Biolegend), DC-LAMP (1:100, rat, DDX0191P-100 Novus) HOPX (1:100, mouse, sc-398703 Santa Cruz), BMAL1 (1:100, rabbit, Cell Signaling). Impress HRP Polymer (Vector laboratories) in combination with AlexaFluor TSA dyes (Thermo Fisher Scientific) was used where TSA based detection was employed. All histological and cytological scoring were performed in a blinded fashion. Numerical codes were used to identify these slides during the scoring. Once all the data were recorded, the identity was unmasked, and final analyses undertaken according to the study group.

Picrosirius red staining. Staining for collagen was performed using the Picrosirius Red Stain Kit (Polysciences Inc.) according to the manufacturer's instructions. Following staining of lung sections, the whole lung was annotated using the color deconvulation macros from Aperio Imagescope for analysis. Quantitation was performed using the Aperio software. Data represent intensity of total section area/ total area in mm^2.

Quantitative PCR:

RNA was isolated/extracted from the inferior lobe of the mouse lung using TRIzol (Life Technologies). RNA was further purified using the RNeasy Mini Elute Clean Up Kit (Qiagen). The quantity and quality of RNA was assessed using the NanoDrop ND- 1000 spectrophotometer (NanoDrop Technologies Inc) and cDNA prepared with TaqMan. SYBR Green gene-expression assays were used to measure mRNA levels for genes of interest. Eukaryotic 18S rRNA (Life Technologies) and 28S (Sigma) were used as an internal control for TaqMan and SYBR Green assays, respectively. The samples were run on a Viia7 real-time PCR thermal cycler (Roche), and the relative ratio of the expression of each gene was calculated using the $2^{\Delta\Delta Ct}$ method. We used a web interface, Nitecap (nitecap.org)(12) to visualize and compare circadian behaviors of *Wnt2*, *Wnt3a, Wnt5a, Wnt5b, Il1b, Fgf7* gene expression from the mouse lung tissues of *Bmal1*$^{creERT2/+}$, and *Bmal1*^{creERT2neg} littermates. Primers: All primers were procured from Thermofisher life sciences. Catalog numbers provided in source file 3.

Western Blot Analysis

Total protein was extracted from lungs harvested and stored at -80°C. Protein was determined by the BCA assay (Thermo Scientific) assay and equal amounts of protein was loaded. 20–50 µg of protein from each sample was resolved on SDS-PAGE gels followed by immunoblotting after PVDF membrane transfer, probed overnight with β-catenin (1:500, 610154 BD Biosciences), β actin (1:3000, ab8227 abcam), and secondary antibodies anti-rabbit HRP-linked IgG (1:5000 7074P2, Cell signaling) or anti-mouse IgG (1:5000 A2228 Sigma) and developed

by chemiluminescence (34579 Supersignal; Thermo Scientific). Relative changes in the expression levels of interested proteins were measured by densitometry analysis using ImageJ 1.47 software and normalized to β-actin.

Statistics

All statistical analyses were performed using GraphPad (Prism V8). For experiments with more than 2 groups and normally distributed data, a one-way ANOVA or two-way ANOVA was performed, and p value adjustments for multiple comparisons were performed using Bonferroni's correction. In experiments where the data was not normally distributed, Mann-Whitney, and Kruskal-Wallis tests were performed. Bioluminescence data traces were analyzed using a modified version of the R script "CellulaRhythm". Statistical data was considered significant if $P < 0.05$. All the plots represent mean of biological replicates, and error bars represent standard error of mean.

Statement on rigor and reproducibility: All studies were performed using animals from either Jackson Labs or animals from in-house breeding. The background strain of each genetically modified animal has been specified and controls were cre negative littermates on that same background. Animals for ScSeq and all the organoid assays were sacrificed between ZT1-ZT2 unless otherwise stated. Reported findings are summarized results from 3-6 independent experiments.

1. G. Yang *et al.*, Timing of expression of the core clock gene Bmal1 influences its effects on aging and survival. *Sci Transl Med* **8**, 324ra316 (2016).
2. H. A. Chapman *et al.*, Integrin alpha6beta4 identifies an adult distal lung epithelial population with regenerative potential in mice. *J Clin Invest* **121**, 2855-2862 (2011).
3. S. Li *et al.*, Foxp1/4 control epithelial cell fate during lung development and regeneration through regulation of anterior gradient 2. *Development* **139**, 2500-2509 (2012).
4. S. Sengupta *et al.*, Circadian control of lung inflammation in influenza infection. *Nat Commun* **10**, 4107 (2019).
5. R. J. Thresher *et al.*, Role of mouse cryptochrome blue-light photoreceptor in circadian photoresponses. *Science* **282**, 1490-1494 (1998).
6. S. L. Zhang *et al.*, A circadian clock regulates efflux by the blood-brain barrier in mice and human cells. *Nat Commun* **12**, 617 (2021).
7. D. B. Frank *et al.*, Emergence of a Wave of Wnt Signaling that Regulates Lung Alveologenesis by Controlling Epithelial Self-Renewal and Differentiation. *Cell Rep* **17**, 2312-2325 (2016).

8. D. B. Frank *et al.*, Early lineage specification defines alveolar epithelial ontogeny in the murine lung. *Proc Natl Acad Sci U S A* **116**, 4362-4371 (2019).
9. S. Ray *et al.*, Phenotypic proteomic profiling identifies a landscape of targets for circadian clock-modulating compounds. *Life Sci Alliance* **2** (2019).
10. W. J. Zacharias *et al.*, Regeneration of the lung alveolus by an evolutionarily conserved epithelial progenitor. *Nature* **555**, 251-255 (2018).
11. A. Butler, P. Hoffman, P. Smibert, E. Papalexi, R. Satija, Integrating single-cell transcriptomic data across different conditions, technologies, and species. *Nat Biotechnol* **36**, 411-420 (2018).
12. T. G. Brooks *et al.*, Nitecap: An Exploratory Circadian Analysis Web Application. *J Biol Rhythms* **37**, 43-52 (2022).

Supplemental Figure 1: Characteristics of UK biobank cohort

A

	Cohort	Hospitalized
N	84823	3015
Male	43.30%	53.90%
Smoking	42.40%	56.30%
Medication	5.50%	11.70%
Prior Case	2.00%	8.80%
Cancer	20.40%	44.70%
Age at Actigraphy (mean±SD)	62.7±7.8	66.7±7.1
FEV1 (mean±SD)	2.9±0.8	2.7±0.8
BMI (mean±SD)	26.7±4.5	27.9±5.1
Death in 30 Days	N/A	12.00%
Age at Hosp. (mean±SD)	N/A	70.4±7.3
Time to Hosp. (mean±SD years)	N/A	3.7±1.9

B

	1st	2nd	3rd	4th	5th
N	16965	16964	16965	16964	16965
Male	51.30%	44.70%	41.20%	39.70%	39.90%
Smoking	47.90%	43.90%	41.90%	40.60%	37.70%
Medication	6.80%	5.70%	5.30%	5.10%	4.50%
Prior Case	3.30%	2.20%	1.60%	1.60%	1.20%
Cancer	22.80%	21.90%	20.30%	19.60%	17.40%
Age at Actigraphy (mean±SD)	63.4±8.0	63.5±7.8	63.0±7.7	62.5±7.7	61.1±7.7
Mean Activity (mean±SD)	21.5±6.9	23.8±5.3	26.4±5.2	29.5±5.4	36.5±8.3
FEV1 (mean±SD)	2.8±0.8	2.9±0.8	2.9±0.8	2.9±0.7	3.0±0.8

C

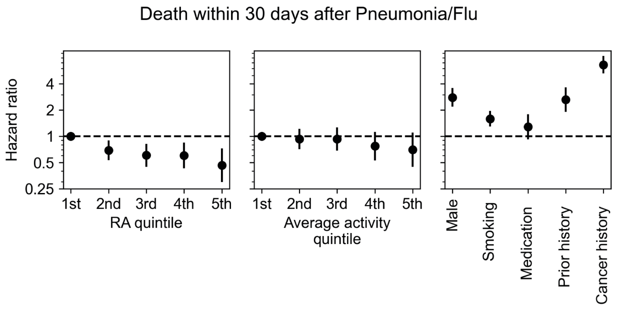

(A) Demographic characteristics of study cohort. (B) Distribution of risk factors across the quintiles of RA scores. (C) Relative Hazards ratio for the common risk factors including age, gender, smoking status (codified as ever smoker or non-smoker), medication use and prior history of hospitalization for respiratory infection and overall activity or exercise (a- referred to as total acceleration on actigraphy measurements)

Supplemental Figure 2: Histopathological analyses after 30 days post IAV infection

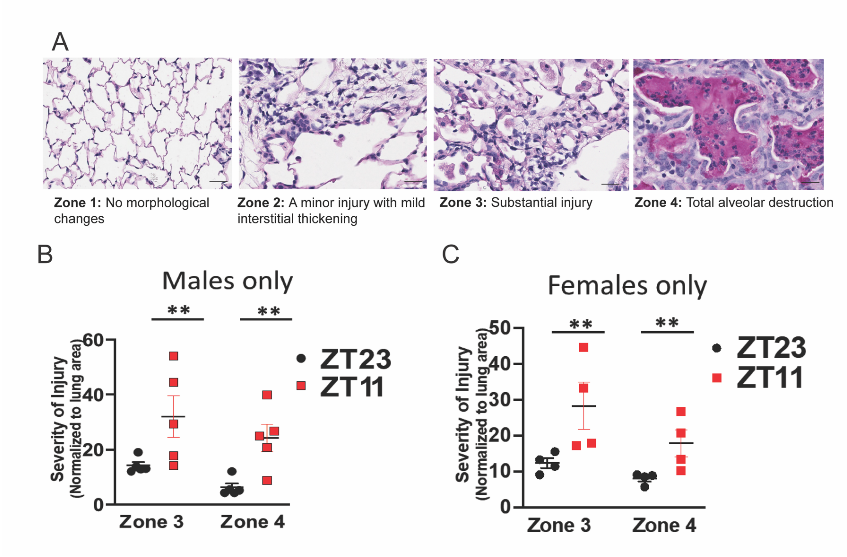

Histological sections were prepared from lungs of C57Bl6J mice infected at either ZT23 or at ZT11 30 days post infection. Representative micrographs are shown in the figures for H&E-stained lung sections on day 30 post-infection depicting (A) Zone 1-minimal injury, Zone 2-minor injury with mild interstitial thickening, Zone 3- severe alveolar injury or Zone 4- complete alveolar destruction. Scale Bar: 50 µm Graphs depecting gender spicifc differences in histopathology 30 days post IAV infection (B) Males (**p=0.001) (C) Females(**p=0.006). The data are represented as mean ± SEM, Two-way ANOVA with Bonferroni's multiple comparisons test.

Supplemental Figure 3: Collagen deposition assays 30 days IAV infection

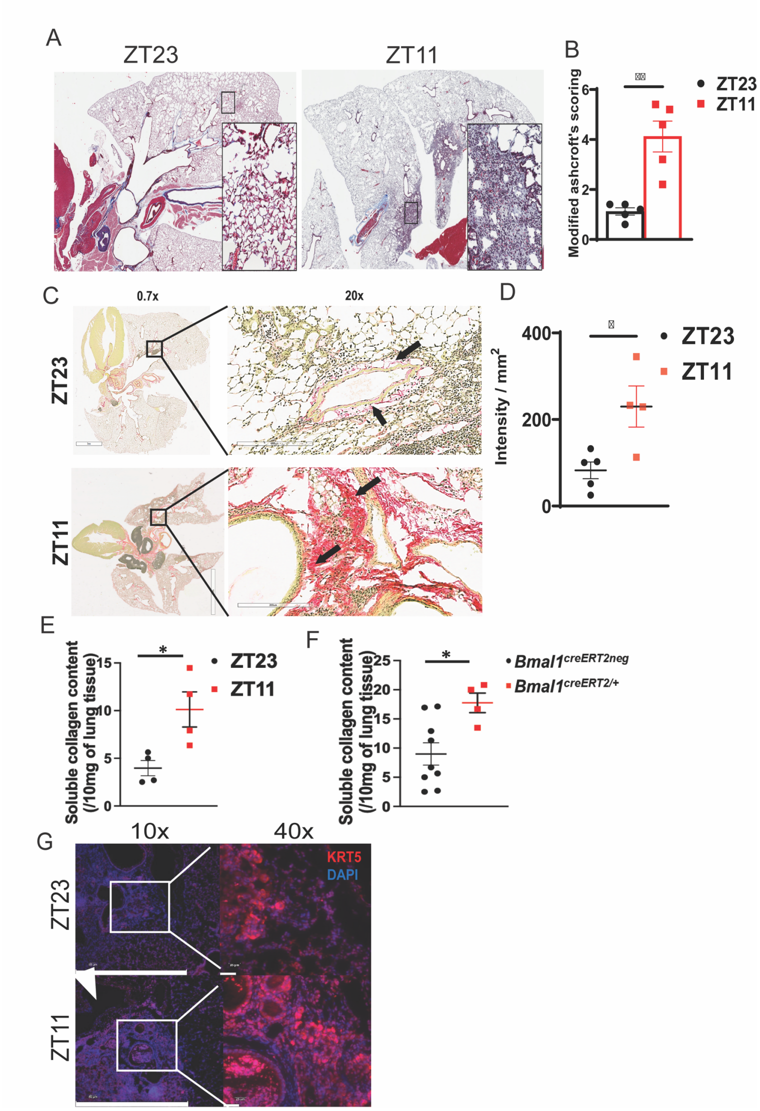

Histological sections were prepared from lungs of C57Bl6J mice infected at either ZT23 or at ZT11 30 days post infection. Representative micrographs for collagen deposition are shown by (A)Trichome staining (photomicrograph bar = 100μm) and (B) summarized statistical analysis, **p=0.006 (C) Sirius red staining showing the deposition of collagen (black arrows). Box is enlarged in the next panel. Scale bar = 3mm and 200 μm (inset) (D) Quantitation performed using the Aperio software. *p=0.04, Soluble collagen in right lung homogenates measured by sircol assay for (E) C57Bl6J mice infected at either ZT23 or at ZT11 (n=4) and (F) *Bmal1*$^{creERT2/+}$ and their Cre^{neg} littermates *infected at CT23 (n=4).* *p= 0.02, *p=0.01. Data are represented as mean ± SEM, n=4-9 mice per circadian time point, Unpaired t test with Welch's correction. (G) Representative KRT5 staining images 30days post infection, scale bar: 50 μm

Supplemental Figure 4: Immunohistochemical analysis after 30 days post IAV infection

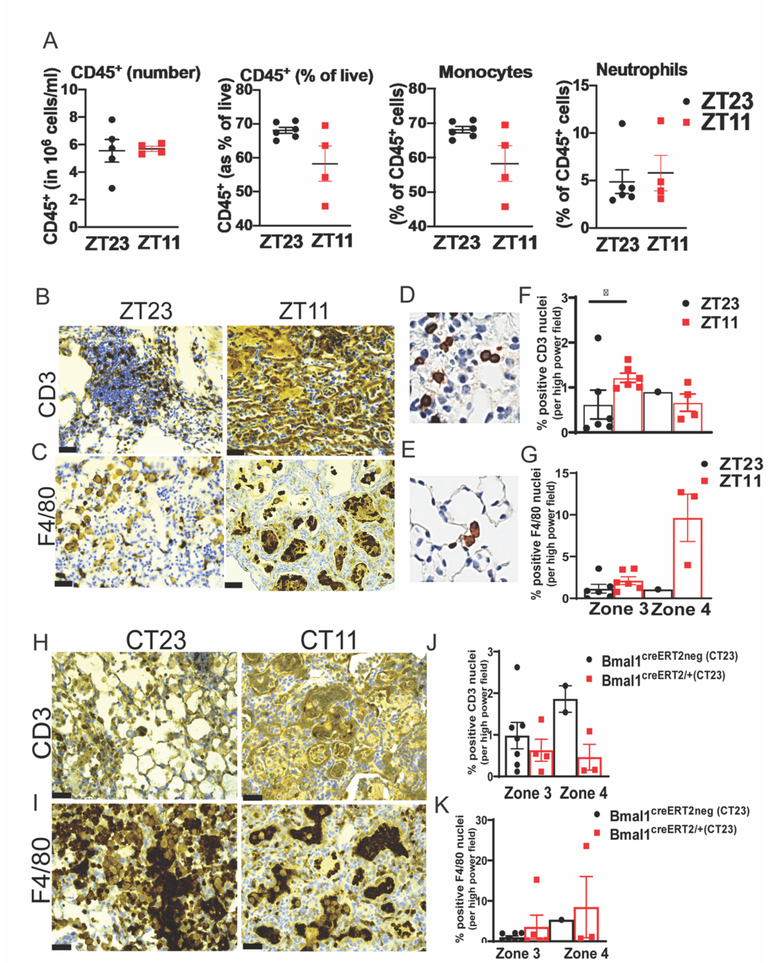

(A) CD45$^+$ cells (as a % of live), CD45$^+$ cell numbers, neutrophils, monocytes (as % of CD45$^+$ cells) in the lung day 10 post-infection (n= 4-6 mice, 2 biological replicates), p>0.05 by Mann-Whitney test. Immunohistochemical staining of (B) lymphocytic infiltration (CD3) and (C) myeloid infiltration (F4/80) in lung tissues of C57Bl6/J mice 30day post IAV infection, the representative images on the right (D) and (E) depicting the cells those were considered positive for respective staining, scale bar: 100 μm. Quantification of IHC images for zone 3 and 4 as defined in Fig 2A above for (F) CD3 (G) F4/80 (n=6-7 mice per circadian time point). *p=0.08, Kruskal-Wallis with Dunn's multiple comparisons test. (H) Immunohistochemical staining of lymphocytic infiltration (CD3) and (I) myeloid infiltration (F4/80) in lung tissues of *Bmal1*$^{creERT2/+}$ and their wild type littermates 30 day post IAV infection, scale Bar: 100 μm. Quantification of IHC images for zone 3 and 4 (J) CD3 and (K) F4/80, p<0.05, Ordinary one-way ANOVA, Tukey's multiple comparison test. Data were pooled from two independent experiments and expressed as mean ± SEM

Supplemental Figure 5: IAV induced lung injury and lung function

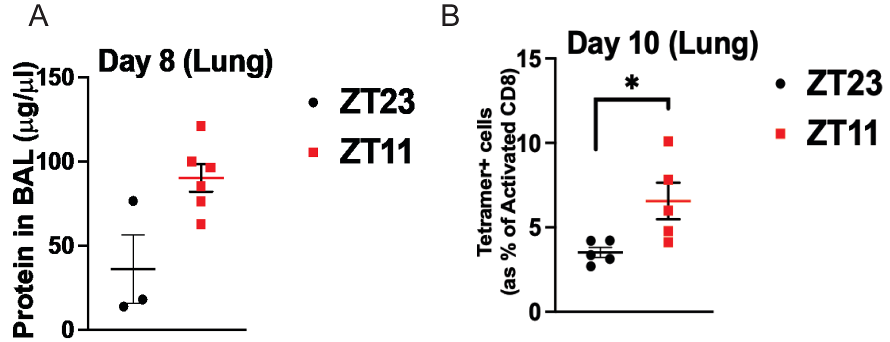

C57Bl6J mice were infected at as depecited in figure 2A. (A) Vascular permeability was reported as total protein content from bronchioalveolar lavage fluid (n=3-6) (B) Virus specific $CD8^+$ T cells in lung tissues 10 days post infection were analyzed by flowcytometry (n= 5), *p=0.01, Unpaired t test, Welch's correction

Supplemental Figure 6: Validation of organoids grown from cells from different levels of respiratory tract

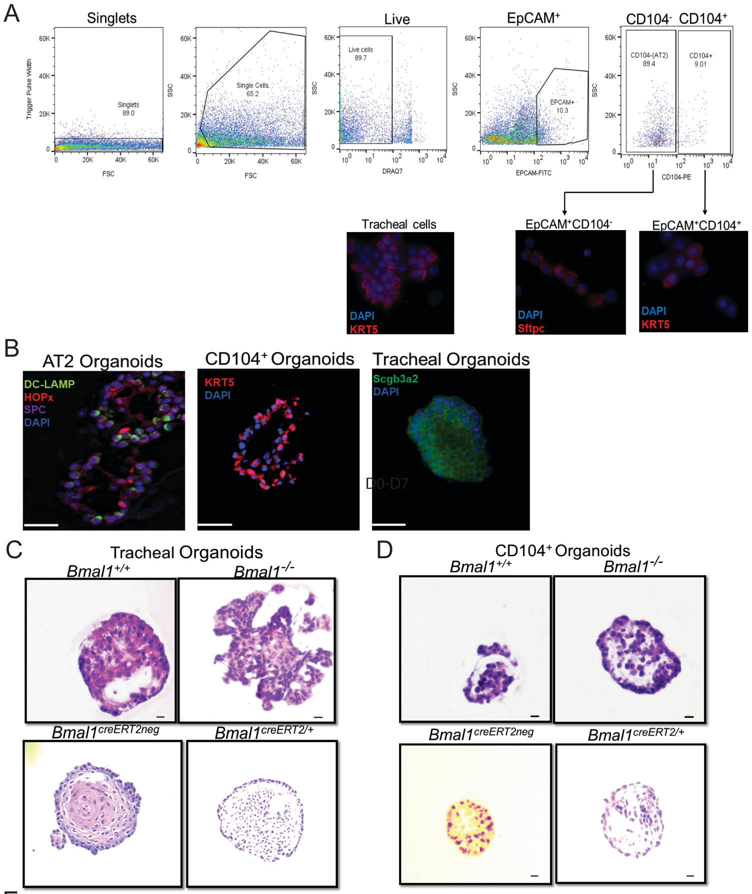

Circadian Characteristics	Tracheal Organoids	CD104+ Organoids	AT2 organoids
Period (h)	26.76±1.0	25.38±0.97	27.34±0.18
Phase	1.8±1.5	9.3±0	7.52±1.0
Amplitude	262242± 182222	27517± 15651	30944± 13721

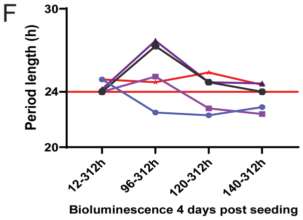

(A) Gating strategy for isolation of CD104$^+$ distal lung airway progenitors and AT2 cells by FACS from *Bmal1$^{-/-}$*, their *Bmal1$^{+/+}$* wild type littermates, *Bmal1$^{creERT2/+}$*, and *Bmal1^{creERT2neg}*. Single cell suspension was sorted for alveolar epithelial cells type II (AT2 as DARQ7$^-$EPCAM$^+$CD104$^-$) and CD104$^+$basal cells (DARQ7$^-$EPCAM$^+$CD104$^+$) post magnetic bead-based depletion of leukocytes and endothelial cells (CD45$^-$CD31$^-$) along with the stained cytospins. (B) Representative immunofluorescence images of AT2, CD104+, and tracheal organoids, scale bar: 50µm. (C) Representative H&E-stained images of embryonic and postnatal *Bmal1* KO tracheal organoids and (D) CD104+ organoids, scale bar:20µm. (E) Summarized circadian

characteristics of PER2::LUC lung organoids expressed as mean±SD (F) Period length(h) of tracheal organoids 4 days post seeding after adding synchronizing agent.

Supplemental Figure 7: Deletion of *Bmal1* reduces regenerative capacity.

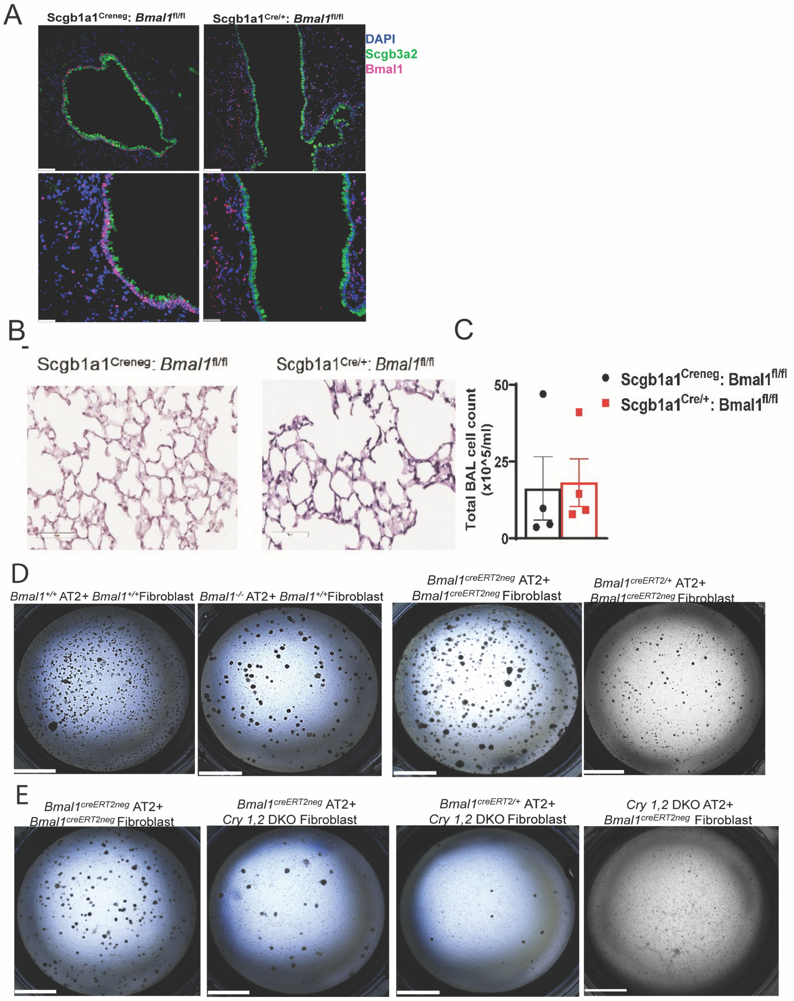

(a) Immunofluorescence staining images to demonstrate the club cell specific loss of Bmal1 in *Scgb1a1*$^{Cre/+}$*Bmal1*$^{fl/fl}$, but not in *Scgb1a1*^{Creneg}*Bmal1*$^{fl/fl}$ littermates, scale bar: 100 µm (B) HE images of lungs and (C) Bronchoalveolar lavage counts from *Scgb1a1*$^{Cre/+}$ *Bmal1*$^{fl/fl}$ and *Scgb1a1*^{Creneg}*Bmal1*$^{fl/fl}$ littermates . Representative images of AT2 organoids that were raised for following combinations, (D) *Bmal1*$^{+/+}$ AT2 with *Bmal1*$^{+/+}$ fibroblasts, *Bmal1*$^{-/-}$ AT2 with *Bmal1*$^{+/+}$ fibroblasts, *Bmal1* ^{creERT2neg} AT2 with *Bmal1*^{creERT2neg} fibroblasts and, *Bmal1*$^{creERT2/+}$ AT2 with *Bmal1*^{creERT2neg} fibroblasts (E) *Bmal1*^{creERT2neg} AT2 with *Bmal1*^{creERT2neg} fibroblasts, *Bmal1*^{creERT2neg} AT2 with *Cry1,2* DKO fibroblasts and, *Bmal1*$^{creERT2/+}$ AT2 with *Cry1,2* DKO fibroblasts, Cry1,2DKO AT2 cells with *Bmal1*^{creERT2neg} fibroblasts . Organoid data were pooled from 3-5 independent experiments with at least 3 technical replicates/experiment expressed as mean ± SEM. Organoid images scale bar: 2000µm

Supplemental Figure 8: Differentially expressed genes in scRNA data

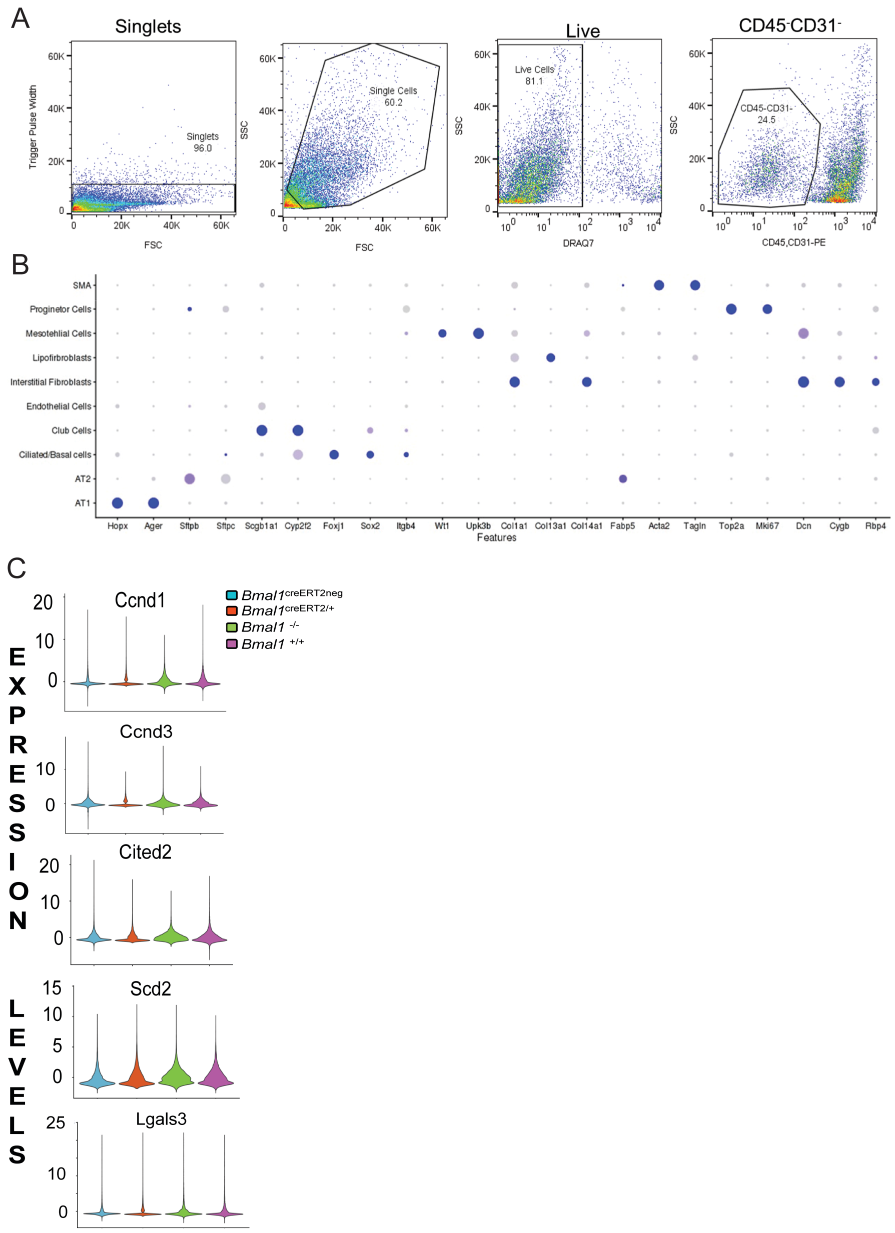

(A)Gating strategy for isolation of lung cells for single cell sequencing from *Bmal1*$^{+/+}$, *Bmal1*$^{-/-}$, *Bmal1*$^{creERT2/+}$, and *Bmal1*^{creERT2neg}. Single cell suspension was sorted on DARQ7^{-}CD31^{-}CD45^{-} lung cells for sequencing. (B) The dot plot showing the percentage of cells expressing the respective selected marker gene based on dot size (C) Violin plots of differentially expressed genes in the *Bmal1* knockout models

Supplemental Figure 9: Ki67 proliferation in cell specific *Bmal1* knockout post infection

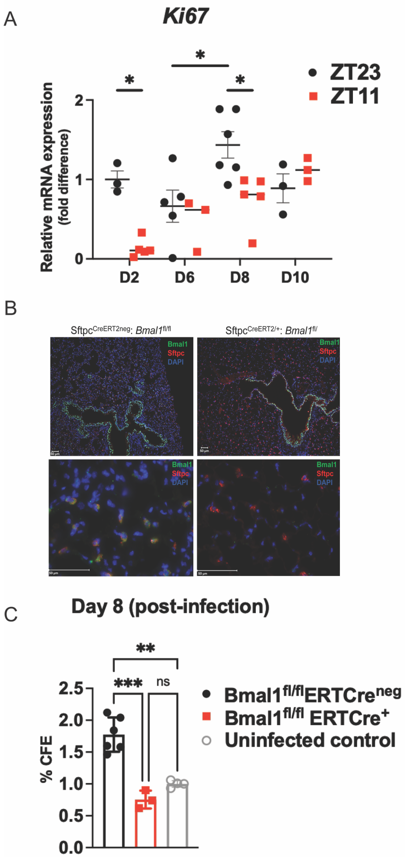

(A) Relative RNA expression of mKi67 RNA by qPCR (*n* = 3-6 mice per day) *=0.017 interaction, Two-way ANOVA with Tukey's multiple comparisons test (B) Representative Immunofluorescence staining images to demonstrate the AT2-specific loss of Bmal1 in *Sftpc*$^{CreERt2/+}$*: Bmal1*$^{fl/fl}$ mice, but not in *Sftpc*^{CreERt2neg}*:Bmal1*$^{fl/fl}$ littermates, scale bar: 50 μm (C) *Bmal1*$^{creERT2/+}$ and their Cre^{neg} littermates were infected at CT23 and lungs harvested 8 days post-infection. AT2 cells were sorted from these mice as well as uninfected Cre^{neg} mice (uninfected controls) and plated for organotypic assays. p<0.0001 by Brown-Forsythe ANOVA test and Dunnett's multiple comparison tests ***p=0.0004 for infected Cre^{neg} versus Cre+; **p=0.0013 for Cre^{neg} infected versus uninfected. CFE plotted after normalization to uninfected control. Data pooled from 2 biological replicates and represented as mean ± SEM.

Supplemental Figure 10: Wnt3a or CHIR did not rescue the *Bmal1* phenotype in AT2 organoids

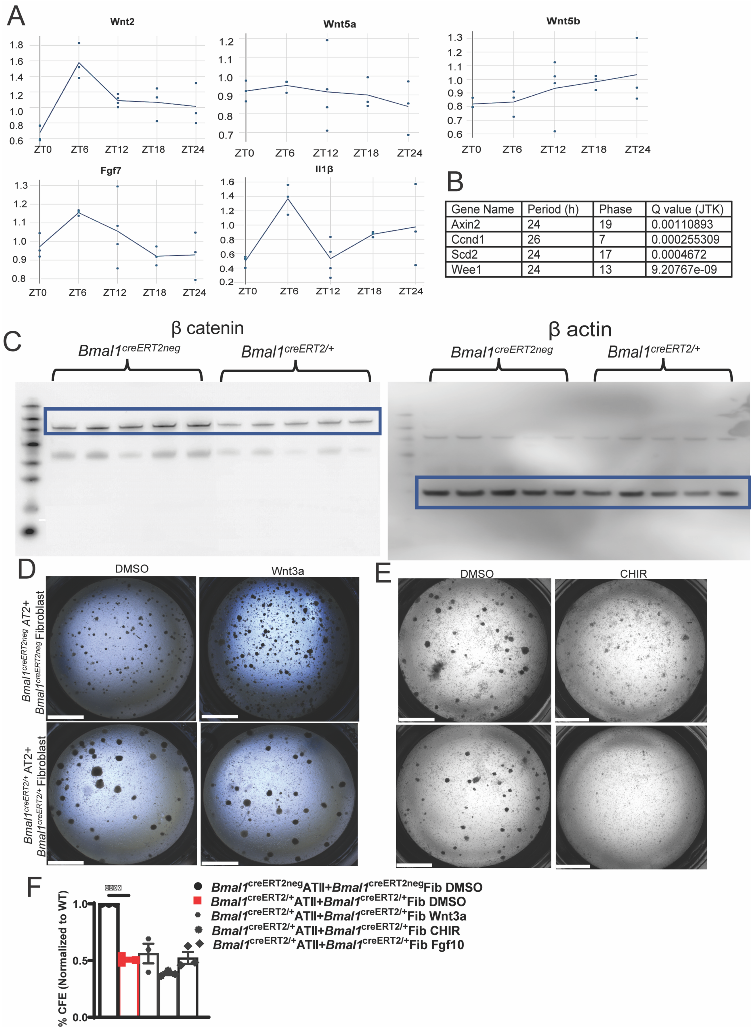

(A) Gene expression of *Wnt2*, *Wnt5a, Wnt5b, Fgf10,* and *Il1β* gens from *Bmal1*^{creERT2neg} lungs harvested at different time intervals determined by qPCR (n = 3-4 per time points). Activation of Wnt signaling by either Wnt3a or GSK-3β inhibitor CHIR-99021 in AT2 organoids. (B) Period and phase of Wnt/ β-catenin targets (http://circadb.hogeneschlab.org/mouse) (C) Immunoblot of β catenin expression from whole lung extracts from *Bmal1*^{creERT2neg} *and* *Bmal1*$^{creERT2/+}$ mice**.** β-catenin (1:500, 610154 BD Biosciences), β actin (1:3000, ab8227 abcam) (D) *Bmal1*^{creERT2neg}AT2 with *Bmal1*^{creERT2neg} fibroblasts, *Bmal1*$^{creERT2/+}$ AT2 with *Bmal1*^{creERT2neg} fibroblasts with either DMSO or wnt3a. (E) *Bmal1*^{creERT2neg}AT2 with *Bmal1*^{creERT2neg} fibroblast, *Bmal1*$^{creERT2/+}$ AT2 with *Bmal1*^{creERT2neg} fibroblasts with either DMSO or Wnt3a. (F) Quantitation expressed as CFE for *Bmal1*^{creERT2neg}AT2 with *Bmal1*^{creERT2neg} fibroblasts, *Bmal1*$^{creERT2/+}$ AT2 with *Bmal1*^{creERT2neg} fibroblasts with either DMSO or CHIR. Organoid data were pooled from 3-5

independent experiments with at least 3 technical replicates/experiment expressed as mean ± SEM. Organoid images scale bar: 2000µm

E; ****p=0.0001, One-way ANOVA, F; **p=0.0087, *p=0.04, Kruskal-Wallis with Dunn's multiple comparisons test.

Tables (as part of other figures):

Tables:

Supplemental Figure 1 (A)

	Cohort	Hospitalized
Male	84913	3031
Smoking	43.30%	54.00%
Medication	42.40%	56.20%
Prior Case	5.50%	11.60%
Cancer	2.00%	8.90%
Age at Actigraphy (mean±SD)	20.40%	44.60%
FEV1 (mean±SD)	62.7±7.8	66.7±7.1
Death in 30 Days	2.9±0.8	2.7±0.8
Age at Hosp. (mean±SD)	N/A	11.90%
Time to Hosp. (mean±SD years)	N/A	70.4±7.3
N	N/A	3.7±1.9

Legend: Demographic characteristics of study cohort.

Supplemental Figure 1 (B)

	1st quintile	2nd quintile	3rd quintile	4th quintile	5th quintile
**N**	16983	16982	16983	16982	16983
**Male**	51.30%	44.70%	41.20%	39.70%	39.90%
**Smoking**	47.90%	43.90%	41.90%	40.60%	37.60%
**Medication**	6.80%	5.70%	5.30%	5.10%	4.50%
**Prior Case**	3.40%	2.20%	1.60%	1.60%	1.20%
**Cancer**	22.80%	21.90%	20.30%	19.60%	17.30%
**Age at Actigraphy (mean±SD)**	63.4±8.0	63.5±7.8	63.0±7.7	62.5±7.7	61.1±7.7
**Mean Activity (mean±SD)**	21.5±6.9	23.8±5.3	26.4±5.2	29.5±5.4	36.5±8.3
**FEV1 (mean±SD)**	2.8±0.8	2.9±0.8	2.9±0.8	2.9±0.7	3.0±0.8

Distribution of risk factors across the quintiles of RA scores.

Supplemental Figure 6(F)

Circadian characteristics	Tracheal Organoids	CD104+ organoids	AT2 organoids
Period (h)	26.7±1	25.38±0.97	27.34±0.18
Phase	1.8±1.5	9.3±0	7.52±0
Amplitude	262242±182222	2517±15651	30944±13721

Summarized circadian characteristics of PER2::LUC lung organoids expressed as mean±SD (F) Period length(h), phase and amplitude of tracheal organoids 4 days post seeding after adding synchronizing agent.

Supplemental Figure 10 (B)

Gene	Period (h)	Phase	Q value (JTK)
Axin2	24	24	0.0110893
Ccnd1	26	26	0.000255309
Scd2	24	24	0.0004672
Wee1	24	24	9.20767e-09

Legend: Period and phase of Wnt/ β-catenin targets (http://circadb.hogeneschlab.org/mouse)
